# Computational Prediction of Biomarkers, Pathways, and New Target Drugs in the Pathogenesis of Immune-Based Diseases Regarding Kidney Transplantation Rejection

**DOI:** 10.3389/fimmu.2021.800968

**Published:** 2021-12-15

**Authors:** Rafael Alfaro, Helios Martínez-Banaclocha, Santiago Llorente, Victor Jimenez-Coll, José Antonio Galián, Carmen Botella, María Rosa Moya-Quiles, Antonio Parrado, Manuel Muro-Perez, Alfredo Minguela, Isabel Legaz, Manuel Muro

**Affiliations:** ^1^ Immunology Services, University Clinical Hospital Virgen de la Arrixaca-Biomedical Research Institute of Murcia (IMIB), Murcia, Spain; ^2^ Nephrology Services, University Clinical Hospital Virgen de la Arrixaca-Biomedical Research Institute of Murcia (IMIB), Murcia, Spain; ^3^ Department of Legal and Forensic Medicine, Biomedical Research Institute (IMIB), University of Murcia, Murcia, Spain

**Keywords:** bioinformatics tool, biomarkers, acute rejection, kidney transplant, new target drugs

## Abstract

**Background:**

The diagnosis of graft rejection in kidney transplantation (KT) patients is made by evaluating the histological characteristics of biopsy samples. The evolution of omics sciences and bioinformatics techniques has contributed to the advancement in searching and predicting biomarkers, pathways, and new target drugs that allow a more precise and less invasive diagnosis. The aim was to search for differentially expressed genes (DEGs) in patients with/without antibody-mediated rejection (AMR) and find essential cells involved in AMR, new target drugs, protein-protein interactions (PPI), and know their functional and biological analysis.

**Material and Methods:**

Four GEO databases of kidney biopsies of kidney transplantation with/without AMR were analyzed. The infiltrating leukocyte populations in the graft, new target drugs, protein-protein interactions (PPI), functional and biological analysis were studied by different bioinformatics tools.

**Results:**

Our results show DEGs and the infiltrating leukocyte populations in the graft. There is an increase in the expression of genes related to different stages of the activation of the immune system, antigenic presentation such as antibody-mediated cytotoxicity, or leukocyte migration during AMR. The importance of the IRF/STAT1 pathways of response to IFN in controlling the expression of genes related to humoral rejection. The genes of this biological pathway were postulated as potential therapeutic targets and biomarkers of AMR. These biological processes correlated showed the infiltration of NK cells and monocytes towards the allograft. Besides the increase in dendritic cell maturation, it plays a central role in mediating the damage suffered by the graft during AMR. Computational approaches to the search for new therapeutic uses of approved target drugs also showed that imatinib might theoretically be helpful in KT for the prevention and/or treatment of AMR.

**Conclusion:**

Our results suggest the importance of the IRF/STAT1 pathways in humoral kidney rejection. NK cells and monocytes in graft damage have an essential role during rejection, and imatinib improves KT outcomes. Our results will have to be validated for the potential use of overexpressed genes as rejection biomarkers that can be used as diagnostic and prognostic markers and as therapeutic targets to avoid graft rejection in patients undergoing kidney transplantation.

## Introduction

The severity and occurrence of rejection in kidney transplant (KT) patients depend on numerous variables that can affect the magnitude and nature of immune responses. Understanding how genetic and molecular factors affect the effector functions of immune cells and donor-specific antibodies (DSA) can better renal stratification receptors based on their immunological risk and thus help the clinician make better decisions to anticipate adverse events (1–5).

In the last two decades, high-throughput technologies such as next-generation sequencing (NGS) and microarrays have been developed. In parallel, international public data repositories have been developed, such as the GEO (Gene Expression Omnibus) database of the National Center for Biotechnology Information (NCBI) (6), the Array Express database of the Institute European Bioinformatics (EBI) (7), in order to store and distribute these data. The large amount of data generated by these technologies makes it necessary to use robust bioinformatics tools that allow us to know *in silico* how molecules interact and regulate the different biological processes that mediate the biological processes of health and disease.

Therefore, functional analysis tools have been developed to explore and identify critical biological processes. These tools are based on biological knowledge databases such as Gene Ontology (GO) or the Kyoto Encyclopedia of Genes and Genomes (KEGG). GO provides a controlled vocabulary of terms (ontologies) to describe gene products in terms of biological processes, cellular components, and associated molecular functions (8). On the other hand, the KEGG is a reference database for the biological interpretation of metabolism and cellular processes (9). Furthermore, the development of genomic and protein databases has led to many bioinformatics tools to predict the properties of proteins and the genome: splice sites, post-translational modifications, stability, pathogenicity. The integration of these new tools and data offers unprecedented opportunities that promise to accelerate and improve our understanding of biology and the search for biomarkers, and specifically for the field of organ transplantation.

The aim was to search for differentially expressed genes (DEGs) in AMR patients and to analyze the types of populations of leukocytes infiltrated in the graft developing diagnostic models using computational predictions in order to find diagnostic and prognostic markers and as a therapeutic target that allows avoiding graft rejection in patients undergoing kidney transplantation.

## Materials and Methods

### Gene Expression Data of Gene Expression Omnibus Repository

For this study, the raw gene expression data studies were downloaded from the Gene Expression Omnibus (GEO) database (6). The characteristics of the GSE cohorts are summarized in **Table 1**. Immunosuppressive treatment in the different GEO studies was similar. Around 40-60% of the transplant recipients in the different studies had triple therapy of corticosteroids + tacrolimus + MMF, while the rest had other combinations in different proportions: corticosteroids + MMF + cyclosporine, tacro + corticosteroids. Regarding induction therapy (thymoglobulin and baxilysimab) there is no information available in the studies. All the bases analyzed in this study have been reviewed and are comparable.

**Table 1:** Characteristics of the gene expression studies of kidney transplantation outcome obtained from the GEO database.

ID Study	Platform	Number of biopsies, n		Total genes	DEGs^a^ (↑/↓)
		NR	AMR		
GSE36059	Affymetrix Human Genome U133 Plus 2.0 Array	281	65	1778	910/868
GSE44131	Affymetrix Human Gene 1.0 ST Array	12	11	2901	1402/1499
GSE50084	Affymetrix Human Gene 1.0 ST Array	20	28	1387	989/398
GSE93658	Affymetrix Human Gene 1.0 ST Array	16	33	2855	1201/1654

DEG, Differentially Expressed Genes; NR, not rejection; AMR, Antibody-mediated rejection; GEO, Gene Expression Omnibus. ^a^DEGs obtained by comparing the NR and AMR groups. ↑: DEG overexpressed in the AMR group. ↓: DEG under-expressed in the AMR group.

### Venn Diagrams

Venn diagrams (10) were used to determine the intersection and analysis of the four analyzed cohorts’ differentially expressed genes (DEGs).

### Functional Analyzes of the KEGG and GO Terms

DEGs in this study were entered into the WebGestalt web tool (6) for the analyzes of Gene Ontology terms (GO) and biological pathways from the English Kyoto Encyclopedia of Genes and Genomes (KEGG) (11). The minimum number of genes to be considered a pathway was adjusted to a value of two.

The Benjamini and Hockberg FDR method adjusted the p-value obtained from each biological pathway with FDR<0.05 considered significant. Only those from the molecular function and biological processes were taken into account for the analyses of GO terms. GO terms with an adjusted p value<0.05 were considered significant (12). Furthermore, the enrichment ratio (ER) is indicated for each GO term and KEGG pathway in this analysis. The ER is defined as the number of genes observed among the number of genes expected for each GO term or KEGG pathway (12).

### Distribution of Leukocytes in Renal Graft Biopsy

The xCell algorithm (13) was used to estimate the relative distribution of leukocytes in graft biopsy, which allows the inference of cell subpopulations from transcriptomic data. This algorithm allows the relative proportion of 64 cell types to be inferred, including lymphoid, myeloid, hematopoietic progenitors, stromal cells, and other cell types, such as epithelial cells or melanocytes. In our study, we focus exclusively on lymphoid and myeloid cells.

**Figure 1:**
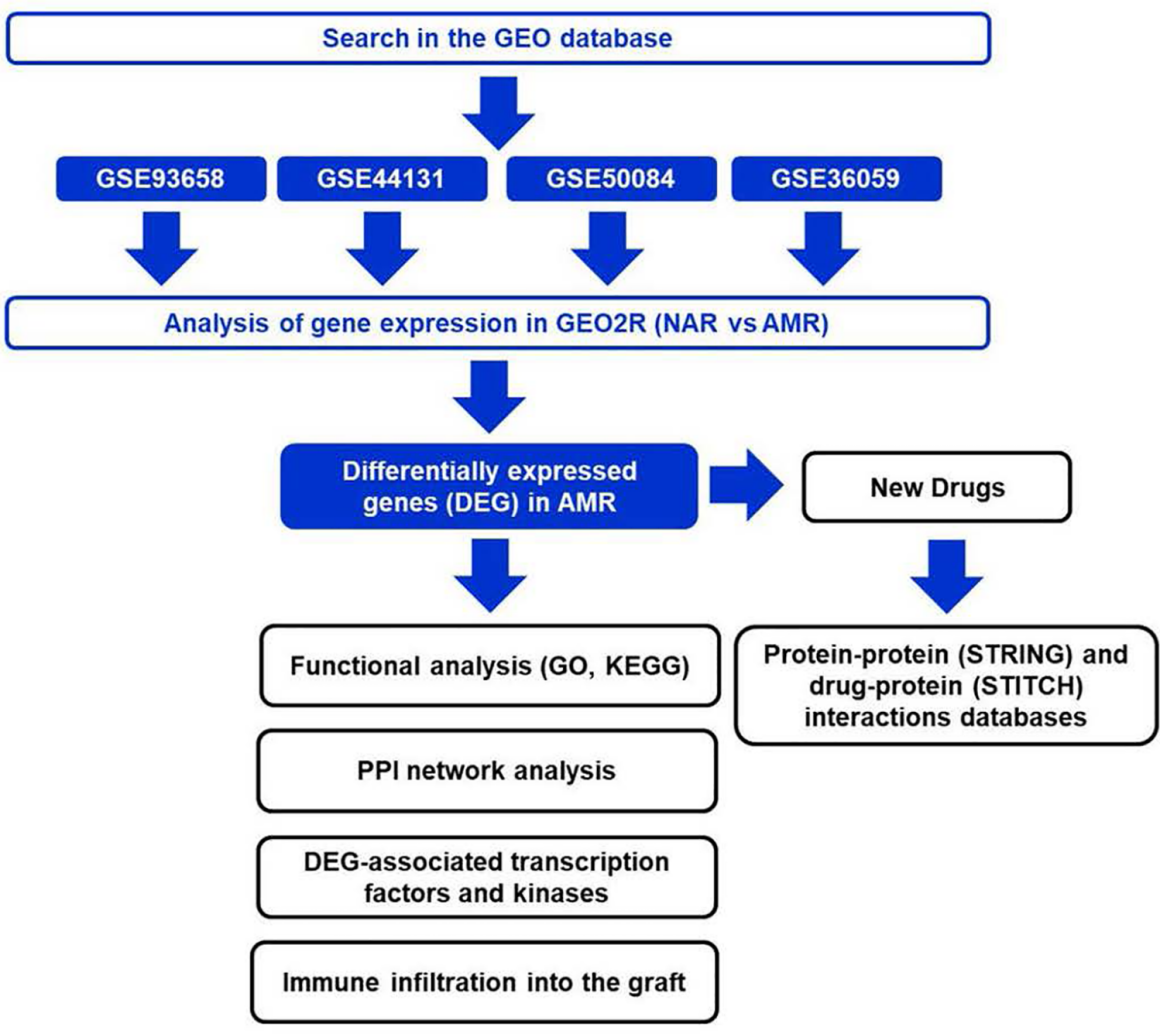
Design of the bioinformatics study applied to antibody-mediated rejection. NAR, No Acute Rejection; AMR, Antibody-Mediated Rejection; GO, Gene Ontology, KEGG, Kyoto Encyclopedia of Genes and Genomes; PPI, Protein-Protein Interaction, GEO; Gene Expression Omnibus, DEG, Differentially Expressed Genes.

The gene expression data of GSE36059, GSE44131, GSE50084, GSE93658 was analyzed using the xCell tool (**Figure 1**), generating a score for each cell population proportional to the relative frequency of that population. The score obtained from each subpopulation and sample will be used later for comparison by traditional statistical methods between transplants with and without AMR.

### Protein-Protein Interaction Networks

The interaction between the proteins produced by the DEGs obtained in the previous sections was used to generate a protein-protein interaction map (PPI) (14) using the Network Analyst web tool (15). This tool uses the Innate DB database of protein interactions.

The nodes represent the proteins in PPI networks, while the junction lines represent the known interactions between the bound proteins. The constructed network was limited to include only the core proteins (zero-order interactions) to avoid the hairball effect, and better visualize the network. The network’s topological properties were evaluated using the betweenness centrality (BC) and the Degree Centrality (GCy). The GI is the number of connections a node has with other nodes, while BC measures the shortest paths that traverse a node. It indicates the degree to which a node is located between other nodes in the network. Nodes with a high BC and DCy index are essential proteins in cell signaling and control information flow.

The modules of the PPI network are tightly connected areas of the network that are considered relatively independent as they often work together to perform a biological function. Therefore, the modules of the PPI networks usually correspond to metabolic pathways or protein complexes. The module explorer of the Network Analyst tool, based on the random-walk algorithm, was used to identify the modules.

Connections within a module and the rest of the PPI network were analyzed. Values ​​of p<0.05 were considered significant. The significance of each module was calculated using the Wilcoxon test. The significant modules were subsequently functionally analyzed using the KEGG and GO term databases. Those GO terms and KEGG pathways with a value of p<0.05 were considered significant.

### Analyzes of Protein Kinases and Transcription Factors

In the search for biomarkers and new therapeutic targets in transplantation, it is crucial to study those genes with altered expression and the entire signaling pathway that regulates them. For this purpose, we employ the Expression2Kinases (X2K) tool that infers the protein kinases and transcription factors that form the regulatory networks upstream of the DEGs obtained previously.

miRNA, B cells were inferred by uploading the list of these genes to the Expression2Kinase (X2K) web tool (15). X2K identifies the transcription factors that regulate DEGs using the ChEA database. Protein kinases associated with transcription factors were obtained through the Kinase Enrichment Analysis module of X2K. Transcription factors and protein kinases with p values <0.05 were considered significant.

### Computational Prediction of New Target Drugs in Overexpressed Genes During AMR Kidney Transplantation

The computational prediction of new target drugs overexpressed genes during AMR kidney transplantation used the Gene2Drug web tool (16, 17).

The Gene2drug algorithm searches for drugs or low molecular weight compounds that induce a significant variation in the expression levels of a list of genes of interest. Gene2drug uses gene expression data from the Connectivity Map database (CMap), containing various cell lines treated with 1309 drugs or low molecular weight molecules. CMap identifies drug-associated transcriptomics alterations. CMap show which genes change their expression induced by these compounds (18). The genes introduced in the Gene2drug algorithm had a value of BC≥100 in the topological analysis of the PPI network, presenting a high connection (**Table S1**). Gene2Drug calculates the English Enrichment Score (ES value) and the p-value corrected by the Benjamini-Hochberg method for the 1309 compounds and drugs of CMap. The ES value ranges from -1 to +1 and represents the degree to which a compound or drug regulates the genes. The ES value uses a generalization of the Kolgomorov-Smirnov method. Compounds with ES values ​​close to +1 will be those that the algorithm predicts will significantly induce the genes of interest, while compounds with values ​​at -1 will be those that will inhibit the genes of interest. Our objective was to search drugs that counteract to focus on those compounds with ES ≤0 values.

### Protein-Protein (STRING) and Drug-Protein (STITCH) Interactions Databases

STRING is a database of protein-protein interactions (16), which includes direct (physical) and indirect (functional) associations obtained from experimental data and computational predictions. STRING version 11.0 was used to obtain the interactions between the PDGFRA and PDGFRB receptors and the proteins with the highest BC and GI values ​​from the PPI network previously analyses (GZMB, STAT1, LYN, IRF1, and IRF8).

STITCH is also a database of computational drug-protein interactions (19). Like STRING, it includes direct (physical) and indirect (functional) associations obtained from experimental data and computational predictions (20). STITCH version 5.0 was used to obtain the drug interactions imatinib.

### Statistical Analysis

The graphs and statistical analyzes were carried out in the Statistical Package for the Social Sciences (SPSS, version 22, Chicago, IL) and GraphPad Prism (version 6, San Diego, CA) software as well as in the R programming language, used for the latter the environment Integrated Development R Studio version 3.4.

The results have been expressed as the mean ± standard deviation (SD) for quantitative data or percentages for categorical data. For the comparison of categorical variables, the χ^2^ test or Fisher’s exact test was used. The verification of the normality of the data was carried out using the Kolmogorov-Smirnov test. The Mann-Whitney U test was used to compare two groups with variables that did not adjust to normality. The Kruskal Wallis test and Dunn’s *post hoc* test with Bonferroni correction for multiple comparisons compared three or more groups. Correlation analyzes were carried out using the Spearman index, as previously published (5, 21, 22). For the longitudinal comparison of two related groups, the Wilcoxon non-parametric test for related samples was used. The Friedman test with Wilcoxon *post hoc* was used to compare three or more related groups. The evaluation of the sensitivity and specificity of a biomarker was carried out by constructing ROC curves. The discriminatory capacity was evaluated using the AUC (**Figure 5**). To obtain the optimal cut-off value that maximizes sensitivity and specificity, we use the Youden index. To correct the p-value in multiple comparisons, the Benjamini-Hochberg or Bonferroni method was used. Values ​​of p<0.05, or p-corrected<0.05 in the case of multiple comparisons, were considered significant for all statistical tests.

## Results

### Description of the Databases Used in the Different Bioinformatic Analyzes

The different databases used in the different bioinformatic analyzes designed to search for biomarkers in KT is shown in **Figure 1**.

Firstly, a search was conducted in the GEO database for studies performed with kidney graft biopsy samples from transplant recipients with AMR or without acute rejection (NR) (**Table 1**). Next, the gene expression data in the GEO2R application to obtain the differentially expressed genes (DEGs) in the AMR samples were analyzed. The DEGs list was used in various bioinformatics analyses to obtain essential characteristics at the molecular and cellular level of the AMR, create diagnostic models, and search for new therapeutic options.

### Differentially Expressed Genes in Biopsies With AMR

The gene expression data of the selected studies (GSE36059, GSE44131, GSE50084, and GSE93658) were analyzed using the GEO2R web tool establishing two groups for each cohort, one with biopsies from transplants NR and AMR.

The GEO2R tool obtained the Fold-change and adjusted p values using the Benjamini and Hochber FDR method, establishing DEGs between the NR and AMR groups with an FDR value <0.05. The number of DEGs obtained for each study is shown in **Table 1**. In the study, a total of 1778, 2901, 1387, 2855 DEGs were obtained in GSE36059, GSE44131, GSE50084, and GSE93658, respectively.

**Figure 2:**
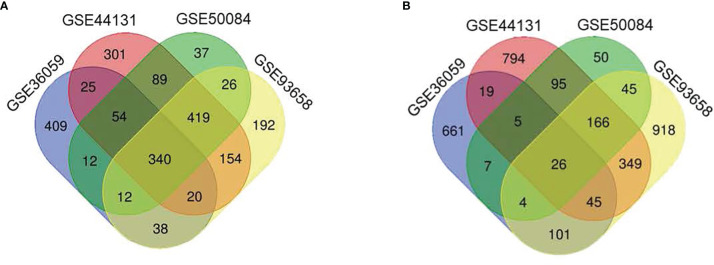
Venn diagrams with the number of differentially expressed genes in four different cohorts analyses. **(A)** The number of genes overexpressed in the antibody-mediated rejection (AMR) group. **(B)** The number of under-expressed genes in the AMR group in each study and the overlap between studies.

We selected those DEGs present in these four studies for further analyses and ruled out genes of little relevance to rejection. In all studies, the deregulation of these genes indicates that they play an essential role in the rejection process and are therefore potential biomarkers. Venn diagram analysis (**Figure 2**) shows 340 overexpressed and 26 under-expressed DEGs in the AMR group. The complete list of DEGs obtained is shown in **Table 2**.

**Table 2:** Total common DEGs in the AMR group in the four cohorts analyzed.

A. over-expressed genes in the AMR group (n=340)								
ADCY7	CSTA	GIMAP1-GIMAP5	ELMO1	PYCARD	MNDA	FAM26F	CD48	HCLS1
BTN3A1	FLI1	IGKC	LYZ	RNASE6	WARS	ACKR1	CLEC4A	UBE2L6
CARD8	EPSTI1	BTN2A2	KLRC4-KLRK1	ARHGAP30	SAMD9L	GIMAP7	SLFN5	CRLF3
CD160	XAF1	MYBL1	ITGAX	ANXA1	CTSS	MARCKS	HLA-DMA	VAMP5
CD300A	RGS18	EMP3	CX3CR1	STAT4	ACSL5	STK4	TNFAIP8L2	ELK3
CD53	CD86	NCKAP1L	GBP5	GRK3	SRGN	LGALS9	PSMB10	TAP1
CIITA	TNFAIP3	STARD4	TLR2	LY86	FCHSD2	PRR5L	APOL3	PELI1
CRTAM	IFI16	CARD16	FUT11	TYMP	PARP14	ADA	RTP4	PSMB9
CST7	SECTM1	FCGR2A	CXCL10	IRF1	ACKR4	ARHGAP9	CD300LF	ISG20
DAPP1	EOMES	NFE2L3	PDCD1LG2	LDLRAD4	SFMBT2	KLRC3	GGTA1P	CXorf36
DDB2	B2M	EBI3	LCP2	PTPRE	MAP3K8	BST2	RNASET2	IL2RB
DPYD	APOL1	CLEC7A	HLA-DOB	IKZF1	GBP2	MICB	CD69	PLEK
DUSP5	IL15RA	TAP2	RARRES1	SAMD3	TLR8	CD5L	AIM2	ADGRE1
EVI2A	PRF1	SP110	ENTPD1	TRIM22	VEGFC	GZMA	MYO1G	PLEK
FCGR1CP//1B//1A	LST1	SAMHD1	RAPGEF5	CDH13	TMC8	PIK3CG	RAB27A	ADGRE1
FCN1	ADGRE2	EFHD2	LCP1	TGM2	HLA-DMB	FMNL3	IRF8	PLEK
FGR	ANKRD22	FGD2	PMAIP1	KLRB1	BATF3	ITGA4	CLEC12A	ADGRE1
FYB	ITGB2	NCF1C//1B	MPEG1	PTGDR	STX11	TNFSF13B	CFP	PLEKHO2
GMFG	TRANK1	TLR7	MLKL	APOL6	IRAK3	TMEM173	MFNG	HLA-DOA
GPR171	MRC1	OAS2	ST3GAL5	C3AR1	PYHIN1	CXCL9	CSF1R	PLEKHO2
GRK5	ARHGAP25	IDO1	SLAMF7	HCP5	LILRA2	LAYN	MYO1F	HLA-DOA
HCST	NFKBIZ	ARHGDIB	SLC15A3	IL2RG	NKG7	TYROBP	PTAFR	PLEKHO2
IFI27	STAT1	GZMB	FCGR3B///FCGR3A	NECAP2	AIF1	C1QB	KLRD1	HLA-DOA
KCNJ2	HCK	TNFRSF25	PATL2	LY96	HCAR3	PRR11	SIGLEC10	CSF2RB
KLF4	GBP4	ANKRD44	P2RX7	FCRL3	RAB31	SH2B3	CD274	APOBEC3G
LILRB2	SLA	BCL2A1	CYBB	ITGAL	FAR2	KLRF1	INPP5D	TNFRSF9
MCUB	CD8A	STK10	SH2D1B	BID	PLA1A	PECAM1	GLIPR1	TSNAX-DISC1
PIK3AP1	PSMB8	GIMAP4	IL1B	ATP8B2	CCSAP	CORO1A	CLIC2	NOS3
PIK3R5	CASP1	XCL1	CELF2	IL12RB1	ARNTL2	GPR65	FGFBP2	TNFRSF1B
PILRA	MALL	HLA-DPB1	FCER1G	CLEC2B	VWF	ICAM1	CHN1	NOS3
PLEKHO1	C5orf56	ADGRE5	IL10RA	GIMAP8	LYST	LYN	RGS10	TNFRSF1B
PRKCB	SIGLEC16	GNLY	CD247	HOPX	THBD	GIMAP2	TLR4	THEMIS2
RASSF5	GIMAP6	SELPLG	WIPF1	BTN3A2	P2RY12	TMEM71	C10orf54	THEMIS2
RCSD1	GNG2	FXYD5	PRKCH	CD55	SLAMF8	CCL8	PLAC8	THEMIS2
RTN1	APOBEC3A	PTGER4	CYTIP	C1orf162	SELE	PSTPIP2	APOBEC3C	BIRC3
RUNX3	LOC153684	PTPRC	ARPC1B	ACTR2	IFIT2	P2RY13	IL7R	CXCL11
SERPINB9	FAM49A	HHEX	SLFN11	ALOX5	HLA-DPA1	S100A4	RNF125	
TAGAP	IL18RAP	LILRA1	CASP4	NCF2	NCF2	NCF2	TM6SF1	
**B. under-expressed genes in the AMR group (n=26)**								
DHRS11	CRABP2	NR0B2	ATP5B	TEF	TMED4	ALDH3A2	POLDIP2	TNPO2
C11orf49	SH3D21	PTDSS2	EPB41L1	ZCCHC14	SLC25A39	CMTM4	SLC25A23	VAPB
C12orf49	HADHB	PART1	ACADS	DNAJC6	ANKRD9	TMEM161A	TIGD5	

AMR, Antibody-mediated rejection; DEGs, differentially expressed genes.

### Biological and Functional Pathway Analyzes of Differentially Expressed Genes

The selected DEGs were subjected to functional analyzes of GO terms and biological pathways of the KEGG. The main biological pathways overrepresented in DEGs are shown in **Table S2A**, where two biological pathways related to AMR stand out, graft-versus-host disease (GVHD; hsa05332, FDR<0.0001) and allograft rejection (hsa05330, FDR<0.0001).

Regarding the under-expressed DEGs (**Table S2B**), only two routes were represented, the fatty acid degradation pathway (hsa00071, FDR=0.0022) and the isoleucine, leucine, and valine degradation pathway (hsa00280, FDR=0, 0022).

The functional analyzes of GO terms for biological processes (BP) and molecular functions (MF) are summarized in **Table 3**.

**Table 3:** The functional analyzes of GO terms for biological processes and molecular functions in DGEs.

A. Over-expressed genes in AMR			
GO ID	Term	ER	*p (FDRa)*
Biological Process (BP)			
GO:0002253	Activation of immune response B	6.51	<0.001(<0.0001)
GO:0002252	Immune effector processes	5.64	<0.001 (<0.0001)
GO:0002443	Immunity-mediated by leukocytes	5.32	<0.001 (<0.0001)
GO:0006955	Immune response	5.62	<0.001 (<0.0001)
GO:0043299	Leukocyte degranulation	5.17	<0.001 (<0.0001)
			
**Molecular function (MF)**			
GO:0001875	Lipopolysaccharide receptor activity	47.46	<0.001 (<0.0001)
GO:0048248	Binding to the CXCR3 receptor	35.59	<0.001 (0.0045)
GO:0008329	Pattern recognition receptor activity	23.73	<0.001 (<0.0001)
GO:0042287	Binding to MHC	14.83	<0.001 (<0.0001)
GO:0001614	Purinergic receptor activity	13.48	<0.001 (0.0028)
			
**B. Under-expressed genes in AMR**			
Biological Process (BP)	Term	ER	*p* (FDR^a^)
GO:0006629	Lipid metabolic process	5.03	0.0001 (0.48)
GO:0019395	Fatty acid oxidation	26.85	0.0001 (0.48)
GO:0034440	Lipid oxidation	26.31	0.0001 (0.48)
GO:0009062	Fatty acid catabolic process	25.30	0.0002 (0.48)
GO:0072329	Catabolic process of monocarboxylic acids	20.72	0.0003 (0.69)
**Molecular function (MF)**			
GO:0016614	Oxidoreductase activity on CH-OH groups	22.63	0.0002 (0.54)
GO:0043878	GA-3P DHase activity	196.1	0.005 (1)
GO:0000253	3-keto sterol reductase activity	196.1	0.005 (1)
GO:0004028	Aldehyde 3 chloroallyl DHase activity	196.1	0.0050 (1)
GO:0016491	Oxidoreductase activity	5.36	0.0055 (1)

GO, Gene Ontology; FDR, False Discovery Rate; BP, Biological Process; MF, Molecular function, ER, Enrichment ratio; ^a^FDR<0.05 were considered significant.

For over-expressed DEGs, the most overrepresented BPs are those related to the immune response activation (GO:0002253, FDR<0.0001) and immune effector processes (GO:0002252, FDR<0.0001). For molecular functions, the most critical terms were pattern recognition receptor activity (GO:0008329, FDR<0.0001) and MHC binding (GO:0042287, FDR<0.0001; **Table 3A**)

GO term with an FDR <0.05 was obtained for under-represented DEGs due to the low number of genes included in the analysis (**Table 3B**). According to the unadjusted p values, the main BPs were lipid metabolism (GO:0006629, p=0.0001) and fatty acid oxidation (GO:0019395, p=0.0001). Regarding the molecular functions, they were the oxidoreductase activity of CH-OH groups (GO:0016614, p=0.0002) and the activity of GA-3P DHase (GO: 0043878, p=0.005).

### Identification of Interaction Networks Between Proteins

**Figure 3:**
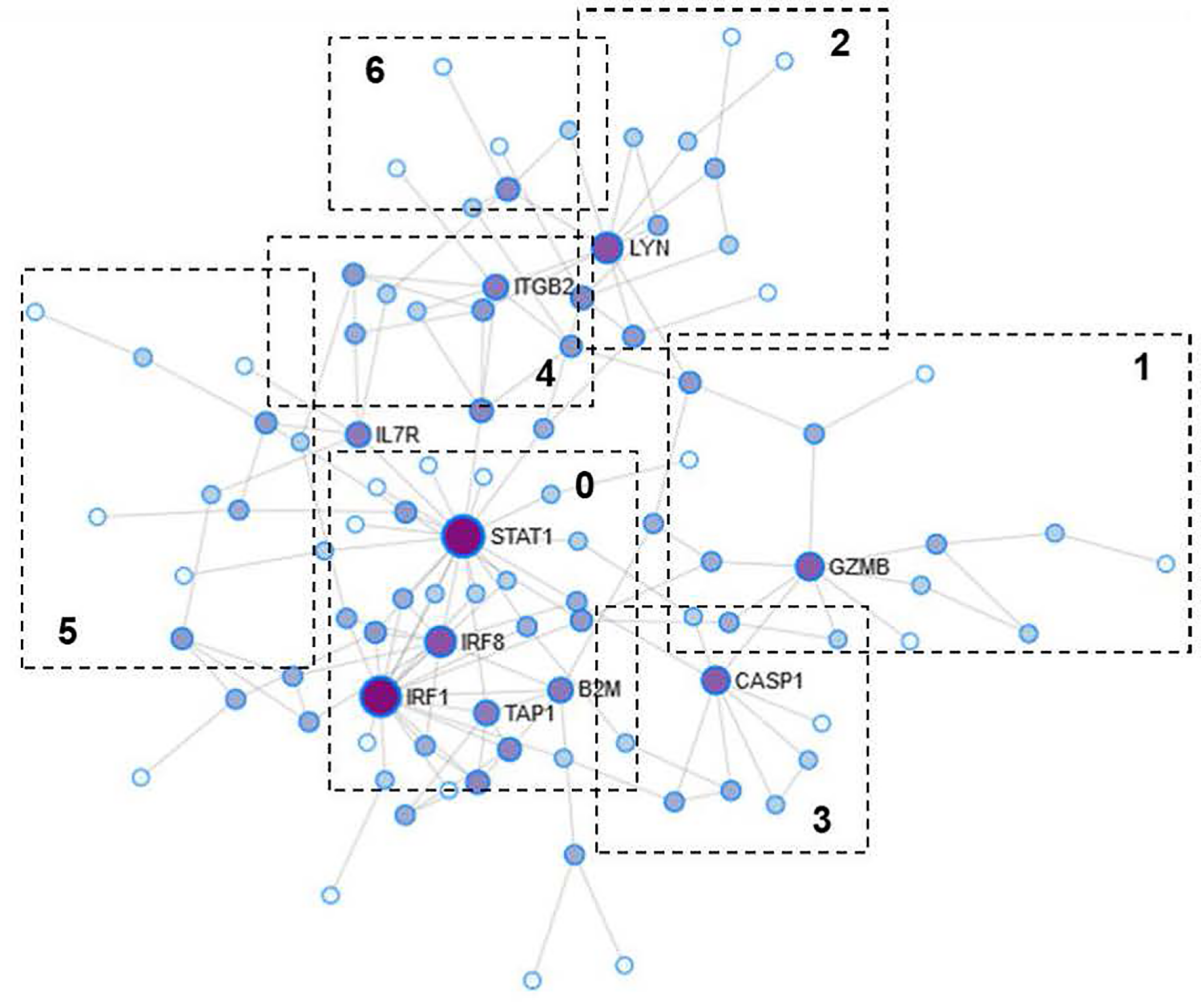
Protein-protein interaction (PPI) network. The size of the nodes represents the degree of interconnection. The color grading represents the centrality of intermediation (BC). Darker colors reflect a high degree of BC. The numbered boxes indicate the functional modules obtained from the analysis of the PPI network.

The PPI network obtained was divided into three subnets, of which we analyzed in greater detail the largest one composed of 98 nodes and 154 interactions (**Figure 3**). The degree of interconnection shows that 25 (25.5%) of the nodes were equal to one, while 73 (74.5%) show a degree ≥2. Of those 73, there are four nodes (STAT1, IRF1, LYN, and IRF8) that show a high GI (≥10): STAT1 (GI=21), IRF1 (GI=19), LYN (GI=10), and IRF8 (GI=10). Regarding the BC, we observe a range from 15 to 2229.9 for a total of 62 (63.2%) of the nodes.

The nodes with the highest levels of BC were STAT1 (BC=2229.9), IRF1 (BC=997.3), LYN (BC=796.9), and GZMB (BC=781.8). The genes encoding the above proteins were among those over-expressed in the AMR group. Of the proteins encoded by genes under-expressed in AMR, only one was represented in the PPI network as a central gene, ATP5B (GI=2, BC=153.7).

The results showed that proteins with a high degree of GI also show high values ​​of BC, which suggests that, possibly, they are the essential proteins in regulating intracellular signaling. Therefore, they can be essential in the search for therapeutic targets or biomarkers.

**Figure 4:**
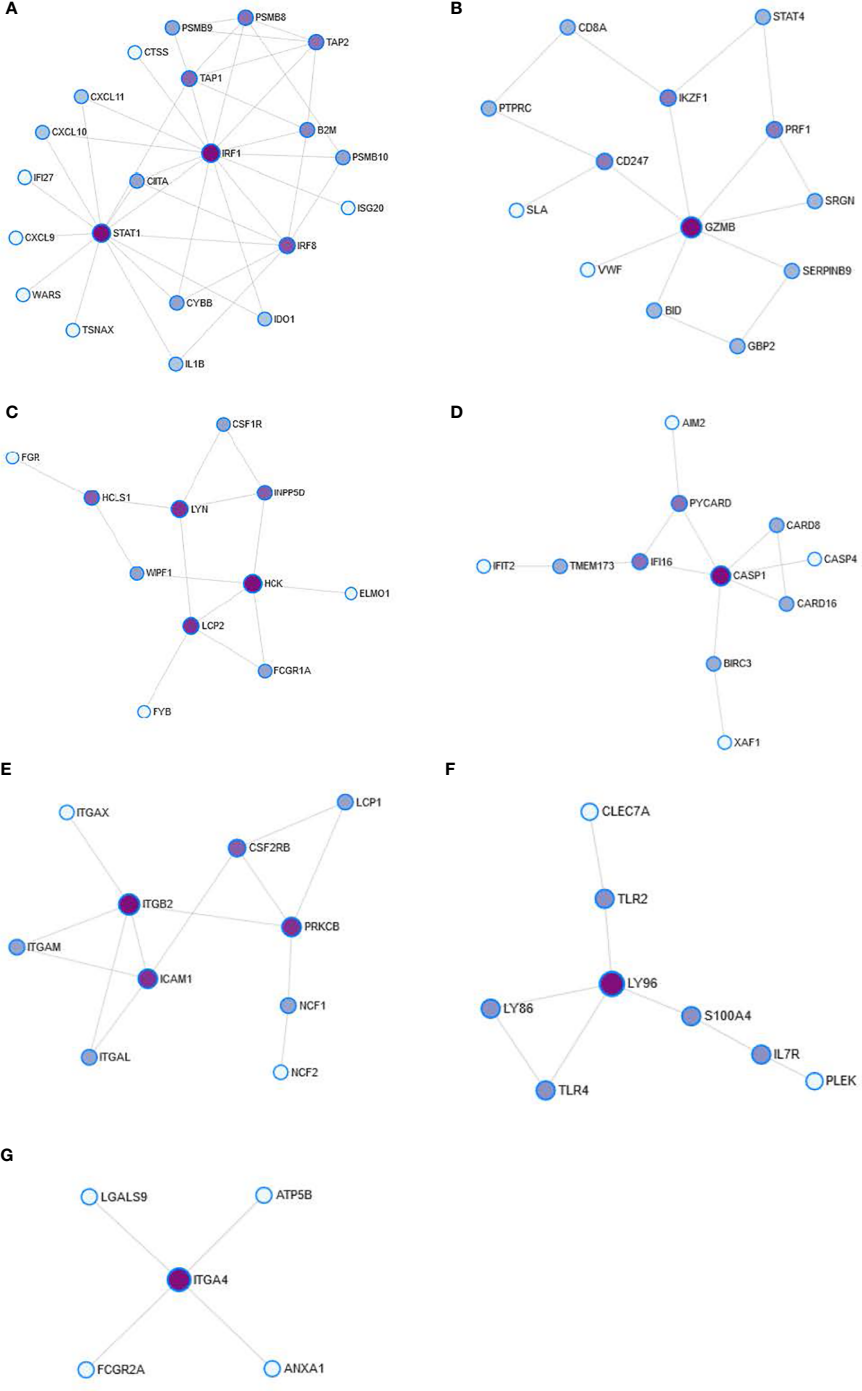
Detail of the modules obtained from the PPI network. The figures represent module 0 **(A)**, module 1 **(B)**, module 2 **(C)**, module 3 **(D)**, module 4 **(E)**, module 5 **(F)**, and module 6 **(G)**. Nodes represent the degree of interconnection. The color grading represents the centrality of intermediation (BC). Darker colors reflect a high degree of BC.

The modules of the PPI network are densely connected areas of the network, which are considered relatively independent since they often work together to perform a biological function. Therefore, the modules of the PPI networks usually correspond to metabolic pathways or protein complexes. In our network, we obtained a total of seven modules (**Figure 3**), although module 6 was not significant (p=0.233) and therefore was not considered for the functional analysis. The proteins of each module are shown in **Table 4**. The graphic representation of the protein connections of each of the modules is shown in **Figure 4**.

**Table 4:** Proteins of different PPI network modules.

Modules	Proteins	*p^a^*
0	B2M, WARS, PSMB10, IDO1, STAT1, TAP1, TAP2, CIITA, PSMB8, IRF1, ISG20, IRF8, CYBB, IL1B, CXCL9, CXCL10, CXCL11, IFI27, PSMB9, TSNAX, CTSS	<0.0001
1	BID, SERPINB9, GBP2, SLA, IKZF1, SRGN, VWF, GZMB, PTPRC, PRF1, CD8A, STAT4, CD247	0.0001
2	HCLS1, ELMO1, HCK, LCP2, FYB, INPP5D, WIPF1, FGR, CSF1R, FCGR1A, LYN	0.0011
3	CASP4, PYCARD, BIRC3, IFIT2, CASP1, CARD8, XAF1, TMEM173, IFI16, AIM2, CARD16	0.0027
4	NCF1, ITGAL, LCP1, ITGB2, ICAM1, ITGAM, PRKCB, CSF2RB, ITGAX, NCF2	0.0034
5	LY96, S100A4, IL7R, TLR4, TLR2, LY86, CLEC7A, PLEK	0.0218
6	ATP5B, ANXA1, FCGR2A, LGALS9, ITGA4	0.2330

a P values <0.05 were considered significant. PPI, protein-protein interaction.

Next, a functional analysis was performed with terms from the KEGG database to obtain the main BPs in each module. The results of the functional analysis of the PPI network modules are shown in **Table 5**. The analysis shows the strong relationship of the modules with effector functions of the immune system such as antigen presentation (Module 0, p=0.0002), NK cell-mediated cytotoxicity (Module 1, p=0.0048), phagocytosis mediated by Fc-γ receptors (Module 3, p=0.0017), and trans-endothelial migration of leukocytes (Module 4, p<0.0001).

**Table 5:** Biological pathways of the KEGG of the different modules.

Modules^a^	Biological routes	FDR^b^
0	Presentation and processing of antigens	0.0002
	Toll-like receptor signaling pathway	0.0004
1	NK cell mediated cytotoxicity	0.0048
	TCR signaling pathway	0.0385
2	Fc-γ receptor-mediated phagocytosis	0.0017
	Chemokine signaling pathway	0.0108
3	NOD-like receptor signaling pathway	<0.0001
	Cytosolic DNA detection pathway	<0.0001
4	Transendothelial migration of leukocytes	<0.0001
	Leishmaniasis	<0.0001
5	Toll-like receptor signaling pathway	0.0047
	Toxoplasmosis	0.0047

a Only the two biological pathways of the KEGG were indicated with the most significant values for each module. Module 6 was not included as there was no statistically significant pathway between the proteins that compose it.

b FDR values<0.05 were considered significant.

### Identification of DEG-Associated Transcription Factors and Protein Kinases

Regarding protein kinases, in **Table S3**, we observed that the most important in the over-expressed genes are MAPK3, CSNK2A1, MAPK14, ERK1, and MAPK1. On the other hand, for the under-expressed genes, the essential protein kinases were DNAPK, CDK1, MAPK14, ERK1, and MAPK1. The MAPK14, MAPK1, and ERK1 protein kinases appear important for over-expressed and under-expressed gene signaling pathways, revealing commonalities upstream of the biological pathways activated during rejection.

For the transcription factors, the most important ones associated with over-expressed genes in the AMR group are SPI, IRF8, RUNX1, RELA, and IRF1, while for under-expressed genes, they were MYC, KLF4, USF1, SOX2, and TCF3 (**Table S4**), showing a clear divergence in pathways between over- and under-expressed genes. The transcription factors SPI and RUNX1 are the ones that target the highest number of DEGs, with 17.9% (61/340) and 17.3% (59/340), respectively, of the total over-expressed DEGs. Regarding the under-expressed DEGs in the AMR group, the transcription factor USF1 targets 23% (6/26) and KLF4 19.2% (5/26) of the total.

### The Cellular Infiltrate in the Graft With Antibody-Mediated Rejection

**Figure 5:**
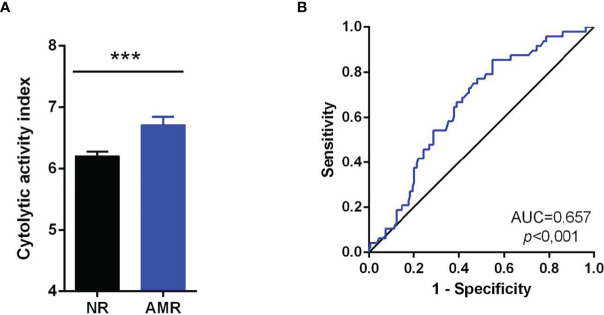
Cytolytic activity index in AMR. **(A)** The cytolytic index (CYT) was compared between the NR group without rejection (NR) and AMR. Comparisons were made using the Mann-Whitney U test. Values expressed as mean ± SEM. **(B)** ROC curve for the diagnosis of AMR using the CYT. As a reference, the non-discrimination diagonal (black line) is represented. The area under the curve (AUC) and statistical significance are indicated on the graph. Values of p<0.05 were considered significant. ***p<0.001. AMR, Antibody-Mediated Rejection. NR, No rejection; AMR, Antibody-Mediated Rejection.

**Figure 6:**
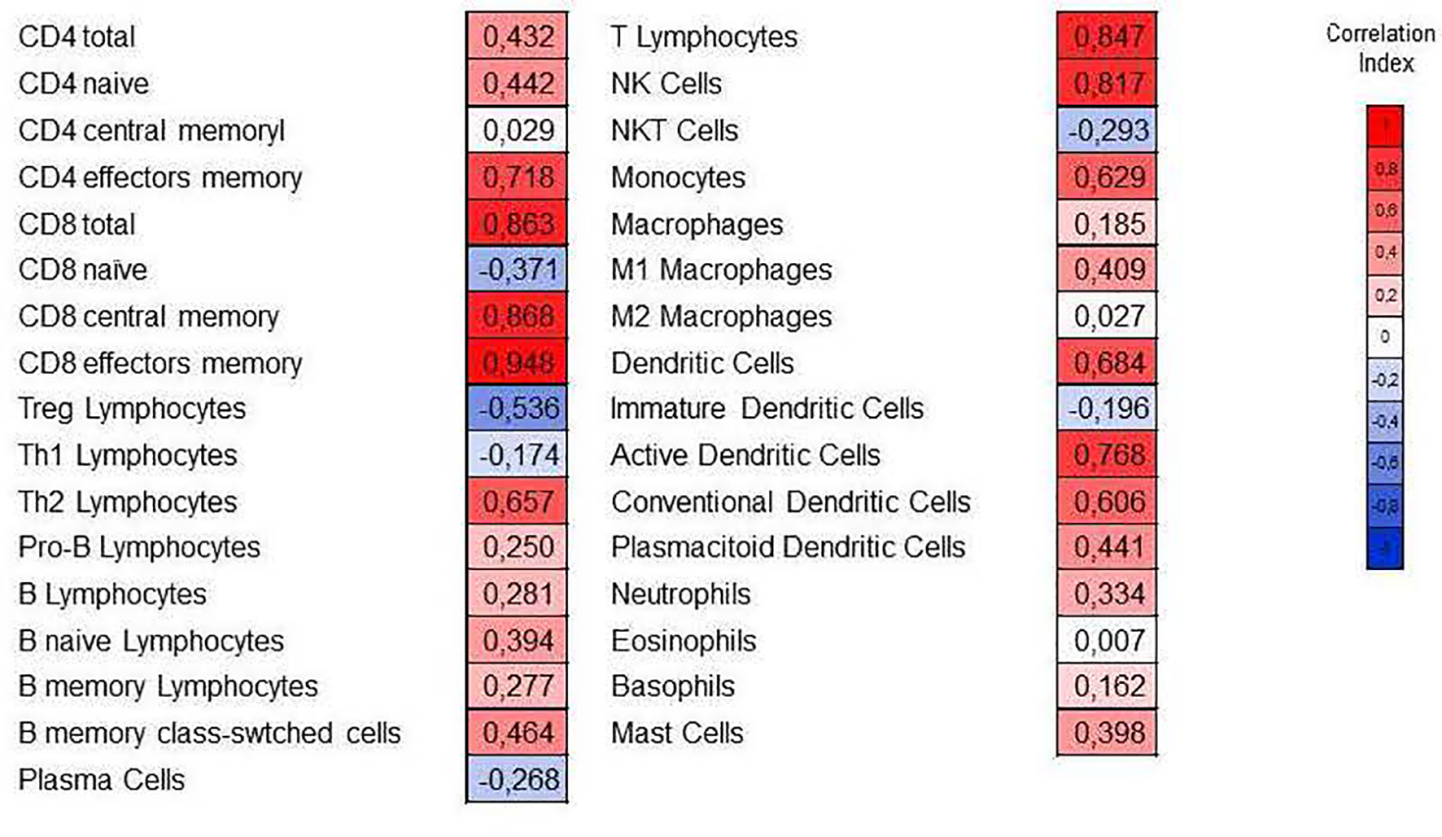
Correlation of the relative abundance of immune subpopulations with the cytolytic index (CYT). The Spearman correlation coefficient is indicated in each cell. The red color indicates a positive correlation, and the blue color indicates a negative correlation.

To evaluate the abundance of immune cells infiltrated in the graft during an AMR, we used the xCell tool (13). This tool is capable of estimating the relative abundance of 64 cell types from transcriptomic data. In our study, we focused on analyzing the 33 immune-type subpopulations that xCell can infer (**Figures 5**, **6**). The transcriptomic data of the cohort GSE36059, composed of 281 biopsies of transplants NR and 65 of transplants with AMR, were entered into xCell. The results obtained of GSE36059 and the rest of the cohorts analyzed were similar are summarized in **Table 6**.

**Table 6:** Analysis of leukocyte population results of the different GSEs using xCell tool.

Leukocyte populations, (mean ± SD)	GSE93658			GSE44131			GSE50084			GSE 36059		
	NR	AMR	*p* (*p*_c_)^a^	NR	AMR	*p* (*p*_c_)^a^	NR	AMR	*p* (*p*_c_)^a^	NR	AMR	*p* (*p*_c_)^a^
CD4 total Lymphocytes	0.047 ± 0.041	0.127 ± 0.075	**<0.001 (0.013)**	0.040 ± 0.045	0.090 ± 0.077	0.071 (1)	0.087 ± 0.064	0.192 ± 0.080	**<0.001 (0.001)**	0.070 ± 0.004	0.070 ± 0.007	0.216 (1.0)
CD4 *naïve* Lymphocytes	0.011 ± 0.015	0.073 ± 0.061	**<0.001 (0.007)**	0.016 ± 0.022	0.065 ± 0.064	0.026 (0.896)	0.038 ± 0.062	0.269 ± 0.050	**<0.001 (<0.001)**	0.010 ± 0.002	0.010 ± 0.002	0.579 (1.0)
CD4 central memory Lymphocytes	0.025 ± 0.036	0.034 ± 0.080	0.652 (1)	0.010 ± 0.012	0.016 ± 0.023	1 (1)	0.010 ± 0.022	0.254 ± 0.038	**<0.001 (<0.001)**	0.001 ± 0.001	0.001± 0.001	0.368 (1.0)
CD4 effectors memory Lymphocytes	0.078 ± 0.055	0.198 ± 0.062	**<0.001 (<0.001)**	0.084 ± 0.048	0.207 ± 0.056	**<0.001 (0.004)**	0.076 ± 0.059	0.337 ± 0.042	**<0.001 (<0.001)**	0.060 ± 0.004	0.080 ± 0.008	0.059 (1.0)
CD8 total Lymphocytes	0.029 ± 0.033	0.154 ± 0.071	**<0.001 (<0.001)**	0.035 ± 0.049	0.124 ± 0.067	0.001 (0.064)	0.059 ± 0.068	0.364 ± 0.061	**<0.001 (<0.001)**	0.069 ± 0.005	0.079 ± 0.007	0.103 (1.0)
CD8 *naïve* Lymphocytes	0.040 ± 0.040	0.025 ± 0.032	0.158 (1)	0.033 ± 0.053	0.039 ± 0.040	0.703 (1)	0.040 ± 0.054	0.041 ± 0.039	0.439 (1)	0.033 ± 0.003	0.031 ± 0.005	0.816 (1.0)
CD8 central memory Lymphocytes	0.012 ± 0.025	0.115 ± 0.067	**<0.001 (<0.001)**	0.031 ± 0.041	0.112 ± 0.060	0.001 (0.067)	0.054 ± 0.061	0.171 ± 0.056	**<0.001 (<0.001)**	0.082 ± 0.006	0.096 ± 0.009	0.127 (1.0)
CD8 effectors memory Lymphocytes	0.028 ± 0.054	0.165 ± 0.084	**<0.001 (<0.001)**	0.052 ± 0.065	0.165 ± 0.040	**0.001 (0.036)**	0.053 ± 0.063	0.435 ± 0.064	**<0.001 (<0.001)**	0.120 ± 0.008	0.161 ± 0.014	0.015 (0.4)
Treg Lymphocytes	0.031 ± 0.027	0.009 ± 0.032	**0.001 (0.047)**	0.021 ± 0.027	0.002 ± 0.008	0.034 (1)	0.042 ± 0.038	0.037 ± 0.024	0.850 (1)	0.06 ± 0.003	0.063 ± 0.006	0.735 (1.0)
Th1 Lymphocytes	0.121 ± 0.051	0.073 ± 0.068	0.011 (0.395)	0.047 ± 0.040	0.032 ± 0.057	0.204 (1)	0.049 ± 0.047	0.013 ± 0.017	0.015 (0.528)	0.048 ± 0.003	0.046 ± 0.007	0.575 (1.0)
Th2 Lymphocytes	0.010 ± 0.024	0.021 ± 0.023	0.079 (1)	0.021 ± 0.024	0.012 ± 0.015	0.422 (1)	0.008 ± 0.021	0.000 ± 0.001	0.142 (1)	0.054 ± 0.002	0.059 ± 0.004	0.283 (1.0)
Pro-B Lymphocytes	0.014 ± 0.014	0.029 ± 0.039	0.306 (1)	0.006 ± 0.009	0.012 ± 0.017	0.434 (1)	0.009 ± 0.011	0 ± 0	**<0.001 (<0.001)**	0.028 ± 0.001	0.026 ± 0.002	0.860 (1.0)
B Lymphocytes	0.015 ± 0.018	0.103 ± 0.074	**<0.001 (<0.001)**	0.028 ± 0.029	0.113 ± 0.072	0.002 (0.070)	0.031 ± 0.042	0.051 ± 0.054	**<0.001 (<0.001)**	0.072 ± 0.005	0.061 ± 0.006	0.461 (1.0)
B *naive* Lymphocytes	0.000 ± 0.001	0.029 ± 0.040	**<0.001 (0.017)**	0.003 ± 0.006	0.031 ± 0.038	0.024 (0.847)	0.005 ± 0.012	0.007 ± 0.017	0.879(1)	0.010 ± 0.002	0.010 ± 0.003	0.299 (1.0)
B memory Lymphocytes	0.003 ± 0.007	0.039 ± 0.042	**<0.001 (0.012)**	0.005 ± 0.012	0.042 ± 0.043	0.004 (0.160)	0.013 ± 0.019	0 ± 0	**<0.001 (0.001)**	0.016 ± 0.002	0.007 ± 0.001	0.131 (1.0)
B memory *class-switched* Lymphocytes	0.047 ± 0.028	0.083 ± 0.050	0.017 (0.610)	0.047 ± 0.030	0.064 ± 0.030	0.324 (1)	0.050 ± 0.031	0.207 ± 0.083	**<0.001 (<0.001)**	0.060 ± 0.004	0.064 ± 0.006	0.295 (1.0)
Plasma Cells	0.029 ± 0.023	0.039 ± 0.042	0.813 (1)	0.038 ± 0.023	0.047 ± 0.044	0.853 (1)	0.030 ± 0.029	0 ± 0	**<0.001 (<0.001)**	0.059 ± 0.003	0.048 ± 0.004	0.092 (1.0)
Tγδ Lymphocytes	0.010 ± 0.021	0.055 ± 0.046	**<0.001 (0.007)**	0.011 ± 0.021	0.039 ± 0.032	0.021 (0.746)	0.025 ± 0.030	0.210 ± 0.037	**<0.001 (<0.001)**	0.033 ± 0.003	0.039 ± 0.005	0.071 (1.0)
NK cells	0.005 ± 0.014	0.042 ± 0.043	**<0.001 (0.003)**	0.004 ± 0.015	0.064 ± 0.040	**<0.001 (0.003**)	0.007 ± 0.015	0.216 ± 0.073	**<0.001 (<0.001)**	0.033 ± 0.004	0.081 ± 0.010	**<0.001 (<0.0)**
NKT cells	0.179 ± 0.092	0.166 ± 0.134	0.376 (1)	0.126 ± 0.066	0.137 ± 0.054	0.711 (1)	0.041 ± 0.041	0.982 ± 0.102	**<0.001 (<0.001)**	0.253 ± 0.010	0.219 ± 0.020	0.095 (1.0)
Monocytes	0.043 ± 0.043	0.147 ± 0.057	**<0.001 (<0.001)**	0.034 ± 0.033	0.171 ± 0.049	**<0.001 (0.001)**	0.010 ± 0.022	0.248 ± 0.041	**<0.001 (<0.001)**	0.023 ± 0.002	0.047 ± 0.006	**<0.001 (0.0)**
Macrophages	0.026 ± 0.046	0.047 ± 0.043	0.009(0.328)	0.016 ± 0.023	0.051 ± 0.025	0.009 (0.339)	0.022 ± 0.029	0 ± 0	**<0.001 (<0.001)**	0.031 ± 0.003	0.028 ± 0.004	0.812 (1.0)
M1 Macrophages	0.001 ± 0.004	0.007 ± 0.019	0.275(1)	0.001 ± 0.002	0.016 ± 0.016	**<0.001 (0.010)**	0.005 ± 0.011	0 ± 0	**0.000 (0.028)**	0.012 ± 0.001	0.012 ± 0.002	0.599 (1.0)
M2 Macrophages	0.138 ± 0.062	0.132 ± 0.059	0.376(1)	0.016 ± 0.017	0.035 ± 0.024	0.039 (1)	0.038 ± 0.026	0 ± 0	**<0.001 (<0.001)**	0.118 ± 0.004	0.111 ± 0.006	0.586 (1.0)
Dendritic cells	0.063 ± 0.043	0.133 ± 0.045	**<0.001 (<0.001)**	0.064 ± 0.031	0.136 ± 0.045	**<0.001 (0.012)**	0.042 ± 0.047	0 ± 0	**<0.001 (<0.001)**	0.079 ± 0.003	0.086 ± 0.005	0.204 (1.0)
Immature Dendritic cells	0.059 ± 0.027	0.061 ± 0.037	0.717(1)	0.026 ± 0.020	0.03 ± 0.034	0.781 (1)	0.016 ± 0.020	0 ± 0	**<0.001 (<0.001)**	0.062 ± 0.002	0.045 ± 0.003	**<0.001 (0.0)**
Active Dendritic cells	0.082 ± 0.057	0.164 ± 0.039	**<0.001 (<0.001)**	0.098 ± 0.053	0.171 ± 0.032	0.001 (0.057)	0.080 ± 0.055	0 ± 0	**<0.001 (<0.001)**	0.144 ± 0.005	0.178 ± 0.006	0.005 (0.1)
Conventional Dendritic cells	0.017 ± 0.026	0.092 ± 0.062	**<0.001 (<0.001)**	0.008 ± 0.014	0.103 ± 0.063	**<0.001 (0.002)**	0.031 ± 0.048	0 ± 0	**<0.001 (<0.001)**	0.049 ± 0.004	0.064 ± 0.007	0.021 (0.7)
Plasmacitoid Dendritic cells	0.027 ± 0.026	0.077 ± 0.035	**<0.001 (<0.001)**	0.005 ± 0.011	0.046 ± 0.019	**<0.001 (0.004)**	0.013 ± 0.012	0 ± 0	**<0.001 (<0.001)**	0.049 ± 0.003	0.058 ± 0.005	0.034 (1.0)
Neutrophils	0.025 ± 0.029	0.083 ± 0.020	**<0.001 (<0.001)**	0.026 ± 0.027	0.090 ± 0.012	**<0.001 (0.002)**	0.044 ± 0.036	0.496 ± 0.022	**<0.001 (<0.001)**	0.420 ± 0.002	0.500 ± 0.003	0.022 (0.7)
Eosinophils	0.067 ± 0.064	0.082 ± 0.154	0.379 (1)	0.032 ± 0.033	0.034 ± 0.078	0.434 (1)	0.029 ± 0.041	0.976 ± 0.126	**<0.001 (<0.001)**	0.116 ± 0.007	0.115 ± 0.015	0.773 (1.0)
Basophils	0.009 ± 0.019	0.110 ± 0.156	**<0.001 (<0.001)**	0.005 ± 0.012	0.101 ± 0.060	**<0.001(0.001)**	0.050 ± 0.052	0.451 ± 0.053	0.548 (1)	0.038 ± 0.004	0.041 ± 0.008	0.937 (1.0)
Mast Cells	0.004 ± 0.004	0.012 ± 0.010	0.015(0.539)	0.001 ± 0.003	0.008 ± 0.009	0.043 (1)	0.004 ± 0.011	0.066 ± 0.009	**<0.001 (<0.001)**	0.011 ± 0.001	0.016 ± 0.001	0.002 (0.0)

NR, no rejection; AMR, Antibody-mediated rejection; ^a^P values obtained by the Mann-Whitney U test. In parentheses is the corrected p-value (pc) using the Bonferroni method for multiple comparisons. Significant results are marked in bold. Values of pc<0.05 were considered significant.

**Figure 7:**
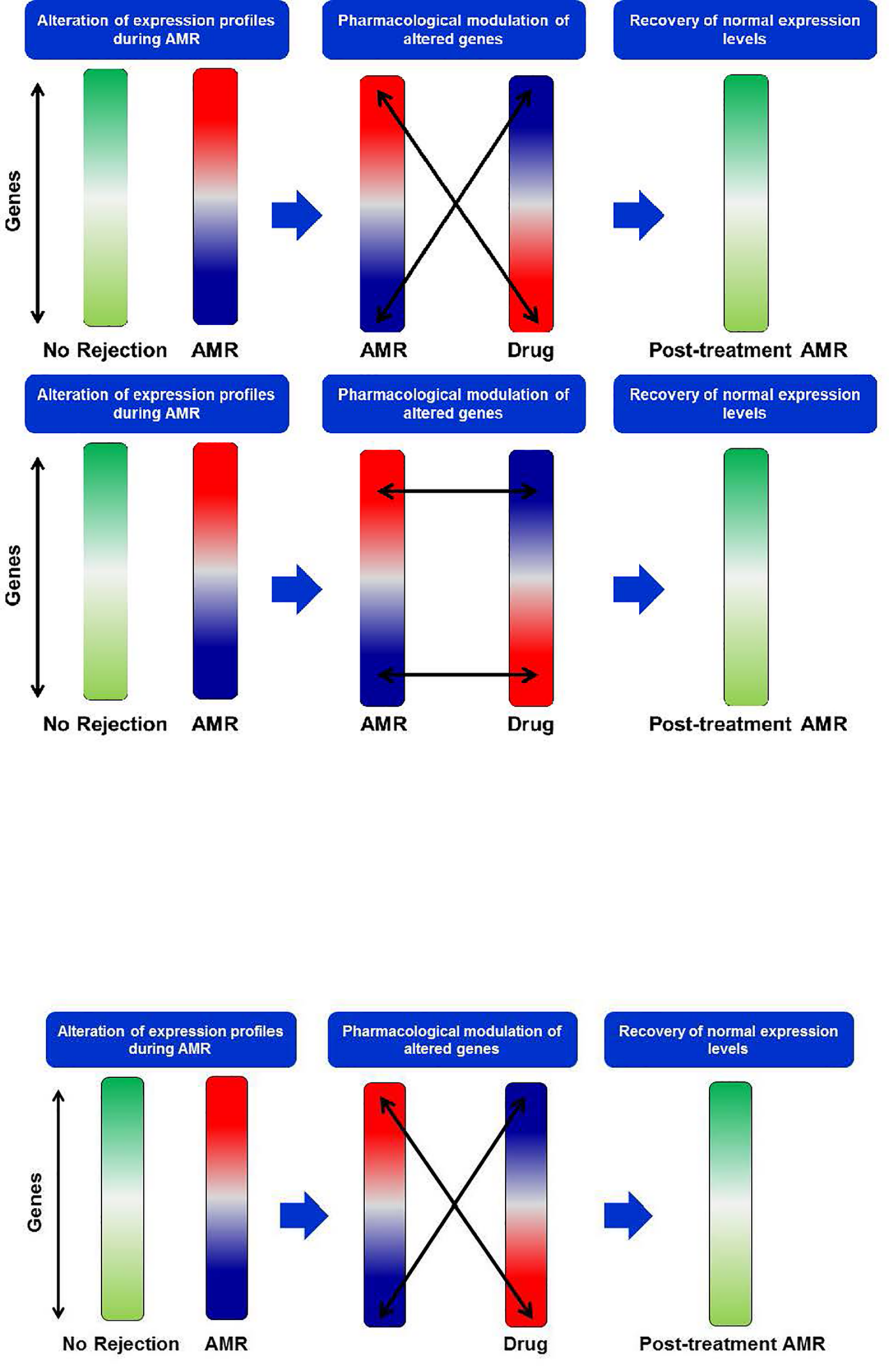
New drug search strategy. During AMR events, an alteration of gene expression profiles occurs. In the search for new drugs, we look for those that induce a transcriptional profile opposite to that observed in AMR to counteract the altered genes and restore normal gene expression levels. AMR, Antibody-Mediated Rejection.

These genes were entered in the Gene2Drug web tool to infer those molecules and drugs with the most remarkable capacity to decrease the expression levels of genes over-expressed in AMR (**Figure 7**). Gene2Drug searches the CMap database, made up of more than 7,000 drug-induced gene expression patterns and low molecular weight molecules. Gene2Drug assigns each drug an ES (Enrichment Score) score ranging from -1 to +1. Those drugs that induce alterations in gene expression similar to those observed in rejection will have ES values ​​close to 1, while producing an alteration in the opposite direction will have values ​​close to -1. For our purpose, we will choose those drugs with ES values ​​closest to -1 since they will be those that best counteract the expression levels of the altered genes in the AMR. The compounds with statistical significance are shown in **Table 7**, and only imatinib is currently approved by the Food and Drug Administration (FDA) and the European Medicines Agency (EMA); that is why we decided to deepen the interactions of this drug.

**Table 7:** Drugs obtained from complete Gene2Drug analysis.

Drugs	ES	*p^a^*
Sulfamonomethoxin	-0.464	<0.0001
PHA-00665752	-0.321	0.0033
Imatinib	-0.320	0.0035
Genistein	-0.317	0.0039
Piperlongumin	-0.310	0.0050
Norfloxacin	-0.309	0.0052
Sulindac sulfide	-0.302	0.0070
Succinylsulfathiazole	-0.293	0.0098
Kaempferol	-0.289	0.0111
Riboflavin	-0.289	0.0113
Rimexolone	-0.288	0.0115
Fluticasone	-0.287	0.0121
Pyretanide	-0.278	0.0165
Dacarbazine	-0.275	0.0180
Bisacodyl	-0.275	0.0181
Parthenolide	-0.271	0.0208
Betanechol	-0.271	0.0209
Tetracicline	-0.256	0.0337
Adipiodon	-0.256	0.0338
Sulfasalazine	-0.256	0.0346
CP-320650-01	-0.252	0.0384
Edrophonium chloride	-0.252	0.0385
Ribavirin	-0.252	0.0390
Sulfafenazole	-0.251	0.0397
Sulmazol	-0.251	0.0404
Methyldopa	-0.250	0.0416
Pheniramine	-0.249	0.0419
Felbinac	-0.247	0.0446
Gabexate	-0.244	0.0492
Todralazine	-0.244	0.0493
CP-863187	-0.244	0.0498

a P values<0.05 were considered significant; ES: Enrichment Score.

**Figure 8:**
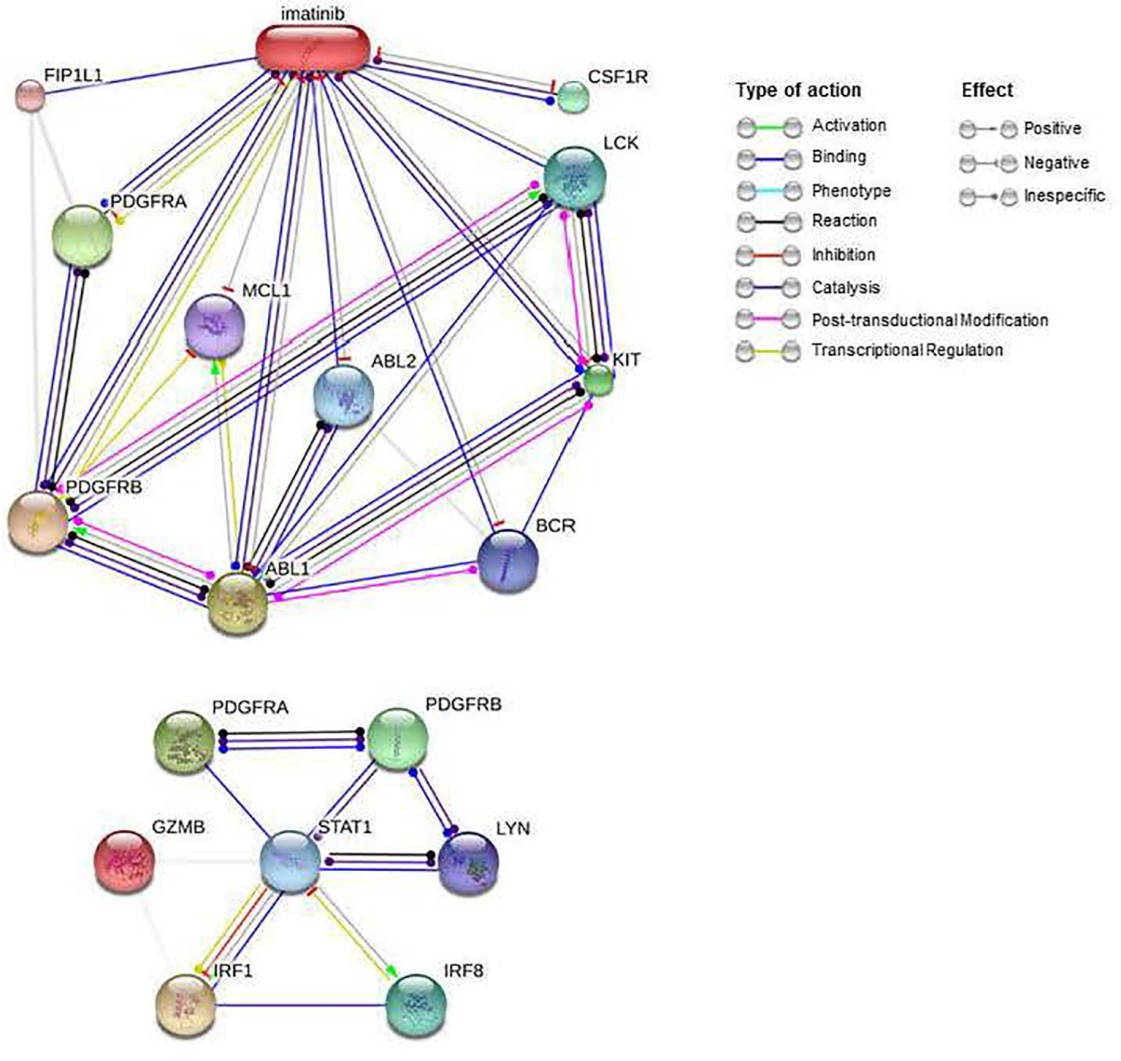
Interactions of imatinib and PDGFRA and PDGFRB proteins. Imatinib interactions were obtained from the STITCH database (Top). PDGFRA and PDGFRB interactions with important genes in the AMR were obtained from the STRING database (below). AMR, Antibody-Mediated Rejection.

Imatinib is an inhibitor of kinases such as the PDGFRA and PDGFRB receptors, currently used to treat some cancers such as chronic myeloid leukemia or gastrointestinal tumors. As we see in **Table 7**, imatinib obtains better ES scores than other drugs commonly used in KT patients, such as Tacrolimus (ES= -0.227), Mycophenolic Acid (ES= -0.180), and Methylprednisolone (ES=0.173). **Figure 8** shows that imatinib acts directly on CSF1R, a receptor for colony-stimulating factor 1, over-expressed in AMR. Furthermore, indirectly, it can alter the expression of STAT1 and LYN through blocking PDGFRA and PDGFRB, which, as we have seen previously, are central proteins that regulate the expression of a large number of genes involved in AMR. Therefore, imatinib may be a promising drug for the prevention or treatment of AMR.

## Discussion

The diagnosis of graft rejection in KT patients is made by evaluating the histological characteristics of biopsy samples. The evolution of omics sciences and bioinformatic techniques has contributed to the development of new diagnostic approaches by combining histopathological characteristics with the gene expression profile of the kidney graft, thereby achieving a more precise diagnosis. It is still necessary to improve our knowledge of the molecular mechanisms underlying kidney rejection to achieve non-invasive diagnostic methods or new therapeutic objectives that improve the clinical management of transplanted patients. For this reason, the central genes and biological pathways that regulate the AMR process were studied to understand better the molecular mechanisms that control it and propose possible biomarkers and new therapeutic options.

Transcriptomics studies have proven to be a valuable tool for understanding the molecular processes that govern graft rejection. One of the main limitations of these studies is, on the one hand, the small number of individuals per study due to the high cost of these techniques. On the other, the variability between different analyzes platforms, challenging to interpret and compare results between different research groups.

Although in some biomedical fields, such as oncology, transcriptomics studies are quite widespread, in transplantation, there are still few investigations with this methodology and, for this reason, a meta-analysis on gene expression is scarce in the literature. In transplantation, the meta-analyses of gene expression carried out have focused mainly on the search for expression profiles associated with graft rejection and tolerance (3, 23), in addition to the search for therapeutic objectives, such as for kidney graft fibrosis (24).

In the context of KT, few studies have focused on studying the specific expression patterns of AMR. Kim et al. (25) performed a meta-analysis where they obtained that CXCL10, CXCL9, and GBP1 were the most over-expressed genes with AMR. Unlike the study carried out by Kim et al. (25), our work has focused exclusively on samples derived from biopsies, excluding those obtained from blood and PBMCs, intending to increase the homogeneity of the study, since the expression profiles in blood and graft may differ, leading to discrepancies between studies, making them difficult to compare.

Our study consisted of four cohorts with 466 renal biopsy samples, 137 from transplants with AMR and 329 from transplants with good graft function. The results showed 340 over-expressed genes in the AMR group, while only 26 were under-expressed. DEGs were functionally classified based on the terms GO for annotation of MFs and BPs, as well as based on the biological pathways of the KEGG.

Genes over-expressed in the AMR group were associated with BPs such as immune response activation, immune effector processes, leukocyte-mediated immunity, and molecular functions such as MHC binding or CXCR3 receptor binding. KEGG metabolic pathway analyzes showed biological pathways directly related to transplantation, such as GVHD and allograft rejection. Increased expression of genes related to MHC binding such as HLA, CD74, or TAP1 genes reflects the antigen-presenting solid activity in the allograft. It is known that in rejection processes, there is an increase in the maturation of antigen-presenting cells (APCs) such as DCs (23), which lead to an increase in the expression of genes related to antigen processing pathways such as CD74 or TAP1 (3, 26).

On the other hand, we have obtained an increase in genes related to the CXCR3 receptor. CXCR3 is a chemokine receptor that polarizes CD4 T lymphocytes towards Th1, promoting infiltration of cytotoxic lymphocytes into the graft. Blocking this receptor is effective in inhibiting macrophage infiltration in acute rejection (27). However, some studies indicate that the infiltration of cells that express CXCR3 is only increased in cell-type rejections and not in humoral ones (28). In our study, the over-expression of the CXCR3 gene was only obtained in two of the cohorts (GSE44131 and GSE93658), which indicates that it is not a good biomarker for AMR.

In contrast, the CXCL9 and CXCL10 genes, which code for CXCR3 ligands, were over-expressed in all cohorts in our analyzes, thus being better indicators of rejection. A possible explanation for this discrepancy may lie in the different abundance of receptor and ligand-producing cells into the graft. While the expression of the CXCR3 receptor is mainly limited to cells of the immune system such as lymphocytes and NK cells (29), the CXCL9 and CXCL10 ligands can be produced in large quantities by epithelial cells of the renal tubule in response to certain stimuli such as IFN-γ (30). Previous studies have shown that the CXCL9 and CXCL10 genes’ over-expression was associated with rejection processes, regardless of the tissue’s type of rejection and origin (3, 31, 32). In heart transplant models, CXCL10 inhibition slows rejection and increases graft survival (33) The data from our analyzes confirm those obtained by other researchers, showing that the ligands that regulate the activation of the CXCR3 receptor are a potential biomarker and therapeutic target. In addition, among the over-expressed genes, we also obtained other biological pathways unrelated to transplantation, such as infection by Leishmaniasis and *Staphylococcus aureus*. These biological pathways are present because the genes involved in the immune response to the allograft are common to those involved in response to infections, such as the HLA or receptor genes of the immunoglobulin Fc fragment (30, 34).

Among the under-expressed genes in the AMR group, the BPs related to the oxidation of fatty acids stand out: catabolic processes of fatty acids, monocarboxylic acids, and lipid oxidation. These results are compatible with those obtained in the analyzes of biological routes of the KEGG where two significantly decreased routes were obtained: degradation of fatty acids and degradation of isoleucine, leucine, and valine, although the differences were not significant after correction for multiple comparisons, probably due to the low number of under-expressed genes obtained in the analyzes. During the activation of the immune system, the proliferation and differentiation processes of lymphocytes lead to metabolic reprogramming. After the lymphocyte activation, there is an inhibition of β-oxidation and an increase in lipid biosynthesis, necessary for cell division (35, 36). These observations are compatible with the results obtained in our study. During allograft rejection, a robust immune response is triggered with increased leukocyte proliferation. This can only be sustained if the metabolic pathways involved in biosynthetic processes are favored, thereby inhibiting the catabolic pathways of essential elements such as lipids and amino acids. Inhibition of lipid synthesis has been shown to interfere with lymphocyte proliferation and slow rejection (37). However, some studies indicate that the microenvironment conditions determine the metabolic status of the effector lymphocytes. In murine models of GVHD, it has been observed that fatty acid oxidation is necessary for effector cell function (38), although most research indicates that carbohydrates remain the primary fuel in immune effector processes (39, 40). This fact becomes evident after immune activation, where the activation of mTORC1 promotes the increase of transporters such as Glut1 to increase the entry of glucose into the cell, necessary to sustain the increase in glycolytic activity. In addition, there is also an increase in other transporters, such as leucine, during lymphocyte activation. Our data suggest that the inhibition of amino acid catabolism, together with the increase in amino acid transporters after immune activation observed in other studies, is a cellular response to maintain the intense biosynthetic activity that occurs during rejection (40, 41). Leucine antagonists or their transporters exert effects similar to those caused after inhibition of mTORC1, altering the differentiation of T cells (41, 42). Our results show that during the processes that mediate AMR, there are changes in immune metabolism compatible with the processes of proliferation and activation of the immune system, and suggest that the pharmacological alteration of the catabolism pathways of some amino acids such as leucine could be a new therapeutic target for the inhibition of lymphocyte proliferation.

Protein-protein interaction networks (PPI) were built to evaluate the relationship between the proteins produced by DEGs and to be able to deduce which are the most important core proteins that control information pathways at the molecular level. The PPI network obtained shows that four proteins control the main biological pathways related to AMR: STAT1, IRF1, LYN, and IRF8. STAT1 is a transcription factor that regulates the expression of genes for cellular response to IFNs and is closely related to the differentiation of T cells towards Th1 (43). The expression of STAT1 has been associated with inflammatory processes in KT related to ischemia-reperfusion damage and BK virus nephropathy (44, 45). Furthermore, STAT1 has been linked to rejection processes in multiple organs in studies conducted in humans (46) and murine models (47). Although STAT1 expression was initially associated with cell-type rejections given its role in Th1 polarization, scientific evidence shows that IFN-related signaling pathways are intimately involved with AMR. That activation of cytotoxic-type effector functions also occurs with intensity in AMR (48). Based on these results, it has been suggested that STAT1 inhibition could be beneficial in slowing down the immune response to graft. Blocking STAT1 with oligonucleotides in murine models of heart transplantation attenuates the recipient’s immune response against the graft, promoting anti-inflammatory effects by inhibiting the expression of MCP-1 (49). Similar results have been obtained in murine models of bone marrow transplantation, where STAT1-deficient mice had decreased IFN-γ production capacity, blocking T cell polarization towards Th1 and reducing rejection events (50).

Furthermore, the STAT1 blockade appears to have more profound effects since it can promote the expansion of Treg (51). However, in other studies, it has been observed that the signaling pathways activated by IFN-γ are also necessary to maintain Treg function, enhancing their ability to prevent rejections. More studies are necessary to understand the true impact of STAT1 blockade for the treatment of rejection.

In this sense, IFN-inducible factors (IRFs, Interferon Regulatory Factor) are transcription factors expressed in response to various stimuli, such as IFN, and have immunoregulatory properties (52). IRF1 is expressed in various organs and various types of immune cells such as NK cells and DCs. IRF1 increases the expression of proinflammatory cytokines such as TNF-α or IL-2 and chemokines such as CXCL10 (30, 53, 54). IRF1 has been shown to act as an early proinflammatory signal during ischemic damage in murine liver transplant models, promoting the secretion of cytokines, such as TNF-α, IFN-γ, or IL-15 (55, 56). Disruption of IRF-1 has been shown to decrease MHC expression in Knock-out (KO) pattern, although all-response rates remained similar to wild-type (57). IRF-1 mediates resistance to necrosis during rejection events, an effect associated with a decrease in IFN-γ (4). In transplant rejection models, the decrease in IRF-1 has been associated with a decrease in the rejection event (58). Other studies carried out with gene expression data obtained from the GEO database have already indicated that the IRF1/STAT1 pathway could be important in rejection processes (46).

On the other hand, the IRF1/STAT1 pathway has also been associated with mechanisms that mediate tolerance in liver transplantation models through the activation of apoptosis (59). On the other hand, IRF8 has a close relationship with B cells differentiation and the secretion of proinflammatory cytokines by the innate immune system (60, 61). IRF8 decreases its expression in liver rejection, although it is a study with a low number of samples (62). In KT, the function of IRF8 has been poorly studied. In a microarray study, IRF8 over-expression has been associated with kidney graft loss in those patients without rejection (63).

Additionally, LYN is a protein kinase of the Src kinase family expressed mainly in hematopoietic cells and played an essential role in regulating the responses of the innate and adaptive immune systems. The study of LYN in the context of KT has not yet been addressed, although the relationship between IFN expression and LYN activation could indicate an important role of the latter in rejection. As we have said previously, IFN pathways appear to be especially active during rejection processes. Plasmacytoid DCs and type I INF-producing APCs increase LYN expression during their activation, acting as a positive regulator in the induction of IFN and the production of cytokines (64). On the other hand, some studies indicate that LYN is involved in the signaling pathways involved in the expression of immunoglobulin Fc receptors such as CD16, or the expression of PIK3CG, a regulator of NK chemotaxis and cytotoxicity, suggesting that the increase in LYN expression could be associated with increased antibody-mediated cytotoxicity (65). Some studies indicate that LYN blockade could prevent the cytotoxic action of NK cells (66). In addition to DCs and NK cells, LYN is also expressed in B cells and is involved in the initiation of BCR signaling and the negative feedback responsible for signal inhibition (67). Inhibitors of this kinase are currently being tested to treat some diseases such as chronic B lymphocytic leukemia (68). Therefore, it could be a possible therapeutic target in transplantation, given its relevant role in activating the B cell and the effector functions of NK cells, although the redundancy of function of the Src family kinases, to which LYN belongs, could limit their activities. In addition, LYN-deficient murine studies produce myeloid hyper-activation, with increased BAFF secretion and a heightened inflammatory state (69). The increase in BAFF has been associated with an increase in the production rate of anti-HLA antibodies. Therefore, the LYN blockade could be counterproductive in the context of KT and should be further studied.

The analysis of transcription factors and protein kinases reveals the importance of IRF1 and IRF8 in regulating genes associated with AMR, like the analysis of PPI networks. However, it also shows the importance of other transcription factors such as RUNX1, RELA, and SPI1. These transcription factors are closely related to the differentiation, proliferation, and activation of cells of the immune system. First, RUNX1 is a hematopoiesis controller that controls the expression of essential genes such as NF-κB and, therefore, inflammatory responses. Second, RELA is the p65 subunit of NF-κB and is essential for its activation (70). SPI1 is a transcription factor of the Ets family expressed in B cells and myeloid cells, crucial in B differentiation and neutrophil activity (71). Bioinformatic analyzes have already reported on the relevance of SPI1 in the regulation of AR processes (70, 72). Thus, PU.1 is a transcription factor encoded by the SPI1 gene and is vital in differentiating and developing macrophages and B cells (73).

Regarding protein kinases, the MAPK family shows a central role in controlling metabolic pathways of both over-and under-expressed genes. The analysis shows that MAPK1 and MAPK14 are important in the expression of genes of both groups. Among the kinases, it is worth mentioning ERK1, a serine-threonine kinase that participates in the Ras-MEK-ERK signaling cascade, involved in multiple processes such as cell adhesion, cell cycle progression, proliferation, and cell survival (72).

During AMR, vasculopathy occurs at the level of the endothelium of the graft vessels. The binding of anti-HLA class I antibodies to the endothelium increases endothelial, smooth muscle proliferation through an ERK1 process, also demonstrated by the generation of anti-non-HLA antibodies such as anti-AT1R (74). The pharmacological blockade of the MEK-ERK pathway effectively reduces chronic allograft nephropathy and the immune response in murine models of KT (75). In cardiac models, ERK inhibition was shown to inhibit the alloresponse, decreasing the leukocyte infiltrate to the graft and IFN production (76). Therefore, ERK1 kinase could be a potential therapeutic target to slow down antibody damage.

The xCell analyzes based on the inference of immune system cells from gene expression data show the important association of NK cell and monocyte infiltration with AMR processes. Therefore, inference data from cell subpopulations infiltrating the graft indicate that NK cells and monocytes are major cellular mediators of graft damage. Therefore, the STAT/IRF molecular pathway acquires great importance since it is directly related to the formation of cytokines, such as IFN and IL-15, or chemistries such as CXCL10, essential for the activation of these cells and their migration towards the graft.

Similar results have been obtained in heart transplantation, where NK cells and monocytes were also associated with AMR (77). Furthermore, several studies carried out in animal transplantation models corroborate the role of these cells in AMR (48, 78). After transplantation, the function of NK cells is exerted through both antibody-dependent mechanisms, such as antibody-dependent cellular cytotoxicity, and non-antibody-dependent mechanisms, through activation by ligands such as osteopontin (79). Although the significance is lost after statistical correction for multiple comparisons, we have observed a clear tendency for patients with AMR to have elevated levels of mast cells. Although mast cells have not been directly related to rejection processes, numerous studies link high levels with fibrosis and chronic graft damage (80, 81). However, its role in transplantation remains controversial since high levels of mast cell-related transcripts have also been found in grafts from tolerant patients (82).

On the other hand, the levels of immature DCs were decreased in patients with AMR. It is known that, during rejection, there is an increase in the antigenic presentation of alloantigens and the maturation and infiltration of APCs (23). Therefore, the maturation of DCs would explain the low relative frequency of immature DCs obtained in our analyzes. In transplantation animal models, inhibition of its maturation has already been shown to suppress graft rejection (83). Therefore, NK cells, monocytes, and DCs are postulated as candidates for their use as biomarkers of AMR. The inhibition of these cells’ maturation and/or the effector functions could be potential therapeutic targets to stop the immune response against the graft effectively.

Despite efforts in searching for new drugs in transplantation, it is still necessary to search for practical strategies for treating or preventing rejection events. In our study, the DEGs derived from the meta-analysis were integrated into bioinformatics applications to suggest new therapeutic options for AMR prevention. Our objective was to find new drugs capable of reversing the expression of the main genes altered during AMR. Of the candidate drugs, only imatinib is currently FDA approved. Imatinib is a tyrosine kinase inhibitor used primarily to treat chronic myeloid leukemia and gastrointestinal tumors (84). With a view to possible use in KT, different studies in murine models have shown the renoprotective effects of imatinib in the long and short term, limiting the progression of glomerulonephritis the prevention of chronic graft nephropathy (85, 86). However, it has not been tested for this purpose in humans. In several clinical cases, imatinib has been used to treat chronic myeloid leukemia after KT has shown to be a safe drug without major side effects (87, 88).

On the other hand, piperlongumine (PL), a natural alkylamide obtained from the *Piper longum* plant, caught our attention, which has recently attracted the attention of researchers due to its immunomodulatory properties. PL is effective in inhibiting STAT3 and inhibiting proliferation progression in breast cancer cell lines (89). It has also been observed to decrease the proliferation and survival of cell lines derived from hematological neoplasms by decreasing the expression of crucial transcription factors such as NK-κB, STAT1, or MYC (90, 91). In autoimmune diseases, PL has also been observed to suppress DC maturation, decrease proinflammatory cytokines, and inhibit NF-κB (92, 93). Therefore, although PL is still in preclinical studies, its immunomodulatory capacities postulate it as a potential drug for use in transplantation. It should be mentioned that, although our study does not cover the full spectrum of available drugs, limiting themselves to 1309 included in the current version of CMap (18), the strategy used is beneficial for identifying new indications for existing drugs. Future studies in animal models and *in vitro* will be necessary to confirm the hypotheses arising in this study.

Our study has several limitations; in the first place, the strategy of identifying DEGs by selecting those statistically significant in all the selected GEO studies generates a bias as they are studies with a very uneven number of samples. While the GSE36059 study included 346 samples, the GSE44131 study only 23 samples, so the statistical power of this study will be lower and will act as a limiting factor when identifying DGEs and other genes of interest that may have been lost. Therefore, other statistical methods are necessary to integrate and normalize the data from different studies. Another aspect of assessing is the type of platform used in the studies. In our case, all the studies were carried out using Affymetrix microarrays. Although the GSE36059 was carried out under another version, the same platform allows the data to be more homogeneous and comparable.

The low number of under-expressed genes in the AMR group implies that the functional and topological bioinformatics analyzes have lower power, so the conclusions should be assumed with caution. Finally, inference studies of the immune populations in the graft show the relevant role of NK cells and monocytes, but these algorithms do not discriminate between cell subtypes. It is known that NK cells, in addition to their cytotoxic function, also possess immunoregulatory functions through the secretion of cytokines and chemokines. Therefore, using other tools such as flow cytometry is necessary to identify further the phenotype of infiltrating NKs in the graft. Another of the shortcomings of the study has been not to include samples from cellular rejections. Future studies with cellular rejection samples will be necessary to determine which expression profiles are common to rejection processes and differentiate between subtypes.

Furthermore, the selected GEO studies do not provide information on the acute or chronic nature of the rejection, which means that the analyzes are based on a heterogeneous cohort regarding the post-transplant period in which graft damage occurs. On the other hand, only samples taken at the time of rejection have been studied. In the future, therefore, it would be interesting to carry out longitudinal studies that make it possible to correlate gene expression profiles in the early stages of transplantation with chronic damage, as other previous own studies (94, 95), which allow us to develop prognostic models for the better classification of patients and thereby improve their clinical management. Finally, the results obtained in silico must be confirmed by *in vivo* and *in vitro* analysis in the future.

In conclusion, in the present study, we have been able to identify the gene expression profile associated with AMR and the central infiltrating leukocyte populations in the allograft through different bioinformatics tools. The functional analysis shows how the processes related to immune activation and antigenic presentation are fundamental in AMR mechanisms. Furthermore, topological analyses using PPI networks show the importance of the IRF/STAT1 pathways of response to IFN in controlling the expression of genes related to humoral rejection. On the other hand, inference analyzes of leukocyte populations show a crucial role of NK cells and monocytes in graft damage during rejection. Future studies will be necessary to validate the results obtained and assess the potential use of over-expressed genes as rejection biomarkers. In addition, the results suggest that the central proteins obtained in the PPI networks could be potential therapeutic targets to improve the results of kidney transplantation.

## Data Availability Statement

The datasets presented in this study can be found in online repositories. The names of the repository/repositories and accession number(s) can be found below: https://www.ncbi.nlm.nih.gov/, 93658; https://www.ncbi.nlm.nih.gov/, 44131; https://www.ncbi.nlm.nih.gov/, 50084; https://www.ncbi.nlm.nih.gov/, 36059.

## Author Contributions

RA, MM, HM-B, and IL participated in designing and contributed to data processing and supervision, optimization, data analysis, and manuscript writing. IL and RA participated in data analysis for gene expression assays and contributed to writing the manuscript. VJ-C, HM-B, JG, CB, MM-Q, and AP participated in the data processing. RA, SL, and MM-P contributed to gene expression data generation, organization, and manuscript writing. AM provided the study samples, participated in discussions in data analysis strategies. All authors contributed to the article and approved the submitted version.

## Funding

Our work was possible thanks to support from Instituto de Salud Carlos III (ISCIII), Spanish Ministry of Economy and Competitiveness. Grant Number PI15/01370, P19/01194, and PI20/00050 and co-funding the European Union with the European Fund of Regional Development (FEDER) with the principle of “A manner to build Europe”.

## Conflict of Interest

The authors declare that the research was conducted in the absence of any commercial or financial relationships that could be construed as a potential conflict of interest.

## Publisher’s Note

All claims expressed in this article are solely those of the authors and do not necessarily represent those of their affiliated organizations, or those of the publisher, the editors and the reviewers. Any product that may be evaluated in this article, or claim that may be made by its manufacturer, is not guaranteed or endorsed by the publisher.

## Abbreviations

ACR, Acute Cellular Rejection; ADCC, Antibody-Dependent Cellular Cytotoxicity; AMR, Antibody-Mediated Rejection; APC, Antigen Presenting Cell; AT1R, angiotensin II type 1 receptor; Betweenness Centrality (BC); cPRA, calculated Panel Reactive Antibody; CTLA4, Cytotoxic T-Lymphocyte-Associated Protein 4; CYT, Cytolytic Index; DC, Dendritic Cell; Degree Centrality (DCy); DEGs, Differentially Expressed Genes; DSA, Donor Specific Antibodies; DV, Decision Values; EMA, European Medicines Agency; ES, Enrichment Score; FDA, Food and Drug Administration; GEO, Gene Expression Omnibus; GO, Gene Ontology; GVHD, Graft-versus-host disease; HLA, Human Leukocyte Antigen; KEGG, Kyoto Encyclopedia of Genes and Genomes; PPI, Protein-Protein Interaction; KT, kidney Transplant; MFI, Mean Fluorescence Intensity; MHC, Mayor Histocompatibility Complex; NGS, Next-Generation Sequencing; NR, Not Rejection; PRA, Panel Reactive Antibody; TCR, T-cell Receptor.
