# Supplementary material for: Computational Prediction of Biomarkers, Pathways, and New Target Drugs in the Pathogenesis of Immune-Based Diseases Regarding Kidney Transplantation Rejection
Source: Front Immunol. 2021 Dec 15;12:800968. doi: 10.3389/fimmu.2021.800968 (PMC8714745; doi:10.3389/fimmu.2021.800968)
Supplement: Supplementary file 1 [file Table_1.docx]

| **Table S1.** Nodes and topological parameters of the IPP network. | | | | | | | | | | |
| --- | --- | --- | --- | --- | --- | --- | --- | --- | --- | --- |
| **Node** | **DCy** | **BC** |  | **Node** | **DCy** | **BC** |  | **Node** | **DCy** | **BC** |
| STAT1 | 21 | **2229.9** |  | IL15 | 3 | 96 |  | ITGAM | 2 | 0 |
| IRF1 | 19 | **997.3** |  | BCL2A1 | 2 | 96 |  | FCGR2A | 2 | 0 |
| LYN | 10 | **796.91** |  | CD86 | 2 | 96 |  | CARD8 | 2 | 0 |
| GZMB | 8 | **781.82** |  | TLR8 | 2 | 96 |  | CXCL10 | 2 | 0 |
| CASP1 | 8 | **644.42** |  | PIK3CG | 2 | 96 |  | CXCL11 | 2 | 0 |
| IL7R | 6 | **573.89** |  | SP110 | 2 | 96 |  | CSF1R | 2 | 0 |
| PTPRC | 4 | **496.67** |  | STK4 | 2 | 96 |  | CARD16 | 2 | 0 |
| ICAM1 | 5 | **494.83** |  | NCF2 | 3 | 86.72 |  | PMAIP1 | 1 | 0 |
| IL1B | 3 | **483.02** |  | LCP1 | 3 | 68.19 |  | WARS | 1 | 0 |
| FCGR1A | 3 | **434.51** |  | XAF1 | 2 | 67.12 |  | SLA | 1 | 0 |
| IRF8 | 10 | **429.2** |  | AIM2 | 2 | 62.7 |  | CASP4 | 1 | 0 |
| B2M | 6 | **408.99** |  | PRKCB | 4 | 55.65 |  | RUNX3 | 1 | 0 |
| CD247 | 3 | **362.67** |  | SERPINB9 | 2 | 46.5 |  | ANXA1 | 1 | 0 |
| STAT4 | 4 | **354.52** |  | S100A4 | 2 | 45.56 |  | ELMO1 | 1 | 0 |
| IL2RB | 4 | **333.64** |  | NCF1 | 2 | 39.05 |  | VWF | 1 | 0 |
| ITGB2 | 6 | **242.47** |  | INPP5D | 3 | 35.37 |  | RASSF5 | 1 | 0 |
| BID | 3 | **236.5** |  | TMEM173 | 2 | 33.38 |  | FYB | 1 | 0 |
| HCK | 5 | **231.33** |  | PYCARD | 3 | 32.97 |  | TLR7 | 1 | 0 |
| IL2RG | 4 | **227.53** |  | LY96 | 4 | 29.23 |  | TYROBP | 1 | 0 |
| CD8A | 3 | **215.48** |  | TLR4 | 3 | 27.48 |  | ISG20 | 1 | 0 |
| LCP2 | 4 | **198.43** |  | LY86 | 3 | 27.48 |  | CLEC7A | 1 | 0 |
| KLRD1 | 3 | **191** |  | IFI16 | 3 | 22.92 |  | CXCL9 | 1 | 0 |
| ITGA4 | 5 | **190.05** |  | WIPF1 | 2 | 21.66 |  | PIK3R5 | 1 | 0 |
| CYBB | 4 | **184.48** |  | TAP2 | 5 | 18.88 |  | ITGAX | 1 | 0 |
| IKZF1 | 3 | **161.12** |  | BIRC3 | 2 | 16.67 |  | IFI27 | 1 | 0 |
| CSF2RB | 4 | **156.18** |  | PSMB8 | 5 | 11.54 |  | FGR | 1 | 0 |
| TAP1 | 6 | **154.5** |  | LGALS9 | 2 | 4 |  | PLEK | 1 | 0 |
| ATP5B | 2 | **153.68** |  | PSMB10 | 3 | 3.48 |  | KLRC3 | 1 | 0 |
| ITGAL | 4 | **137.29** |  | GBP2 | 2 | 1.5 |  | MARCH1 | 1 | 0 |
| PRF1 | 3 | **134.98** |  | CIITA | 3 | 0 |  | IL15RA | 1 | 0 |
| 7HCLS1 | 3 | **127.5** |  | PSMB9 | 3 | 0 |  | TSNAX | 1 | 0 |
| TLR2 | 3 | **108.71** |  | IDO1 | 2 | 0 |  | CTSS | 1 | 0 |
| IFIT2 | 3 | **104.46** |  | SRGN | 2 | 0 |  |  |  |  |
| IPP: Protein-protein interaction networks; BC. Centrality of intermediation; DCy. Degree of interconnection; Proteins with values of BC≥100 are highlighted in bold. The genes that encode these proteins were those selected for the analysis of prediction of new drugs carried out using the Gene2Drug application (1). | | | | | | | | | | |
