# Supplementary material for: Computational Prediction of Biomarkers, Pathways, and New Target Drugs in the Pathogenesis of Immune-Based Diseases Regarding Kidney Transplantation Rejection
Source: Front Immunol. 2021 Dec 15;12:800968. doi: 10.3389/fimmu.2021.800968 (PMC8714745; doi:10.3389/fimmu.2021.800968)
Supplement: Supplementary file 2 [file Table_2.docx]

| **Table S2.**  Analysis of leukocyte population results of the GSE44131, GSE50084, GSE93658 using xCell tool. | | | | | | | | | | | | | | | | | | | | |
| --- | --- | --- | --- | --- | --- | --- | --- | --- | --- | --- | --- | --- | --- | --- | --- | --- | --- | --- | --- | --- |
|  | **GSE44131** | | | |  |  |  | **GSE50084** | | | |  |  |  | **GSE93658** | | | |  |  |
|  | **NR** | | **AMR** | |  |  |  | **NR** | | **AMR** | |  |  |  | **NR** | | **AMR** | |  |  |
| **Leukocyte populations** | **Media** | **Desviacion** | **Media** | **Desviacion** | **p value** | **pc** |  | **Media** | **Desviacion** | **Media** | **Desviacion** | **p value** | **pc** |  | **Media** | **Desviacion** | **Media** | **Desviacion** | **p value** | **pc** |
| B-cells | 0.0283 | 0.0292 | 0.1139 | 0.0724 | 0.0021 | 0.0708 |  | 0.0317 | 0.0423 | 0.0519 | 0.0548 | 0.0000 | **0.0000** |  | 0.0153 | 0.0184 | 0.1031 | 0.0746 | 0.0000 | **0.0002** |
| Basophils | 0.0050 | 0.0122 | 0.1012 | 0.0600 | 0.0000 | **0.0017** |  | 0.0502 | 0.0527 | 0.4514 | 0.0531 | 0.5480 | 1.0000 |  | 0.0090 | 0.0193 | 0.1102 | 0.1564 | 0.0000 | **0.0008** |
| CD4+ T-cells | 0.0404 | 0.0450 | 0.0902 | 0.0772 | 0.0718 | 1.0000 |  | 0.0872 | 0.0647 | 0.1921 | 0.0809 | 0.0000 | **0.0011** |  | 0.0475 | 0.0417 | 0.1271 | 0.0750 | 0.0004 | **0.0136** |
| CD4+ Tcm | 0.0108 | 0.0130 | 0.0163 | 0.0233 | 1.0000 | 1.0000 |  | 0.0105 | 0.0226 | 0.2542 | 0.0384 | 0.0000 | **0.0000** |  | 0.0253 | 0.0367 | 0.0346 | 0.0807 | 0.6525 | 1.0000 |
| CD4+ Tem | 0.0842 | 0.0489 | 0.2077 | 0.0564 | 0.0001 | **0.0046** |  | 0.0765 | 0.0597 | 0.3371 | 0.0430 | 0.0000 | **0.0000** |  | 0.0785 | 0.0553 | 0.1990 | 0.0621 | 0.0000 | **0.0001** |
| CD4+ memory T-cells | 0.0766 | 0.0712 | 0.1593 | 0.1106 | 0.0648 | 1.0000 |  | 0.1108 | 0.0820 | 0.2878 | 0.1263 | 0.0000 | **0.0003** |  | 0.0707 | 0.0668 | 0.2049 | 0.1136 | 0.0001 | **0.0046** |
| CD4+ naive T-cells | 0.0161 | 0.0226 | 0.0660 | 0.0640 | 0.0264 | 0.8961 |  | 0.0390 | 0.0630 | 0.2693 | 0.0503 | 0.0000 | **0.0000** |  | 0.0118 | 0.0158 | 0.0734 | 0.0620 | 0.0002 | **0.0077** |
| CD8+ T-cells | 0.0358 | 0.0500 | 0.1245 | 0.0675 | 0.0019 | 0.0649 |  | 0.0591 | 0.0683 | 0.3641 | 0.0616 | 0.0000 | **0.0000** |  | 0.0296 | 0.0332 | 0.1544 | 0.0719 | 0.0000 | **0.0000** |
| CD8+ Tcm | 0.0310 | 0.0415 | 0.1123 | 0.0610 | 0.0020 | 0.0675 |  | 0.0543 | 0.0615 | 0.1712 | 0.0566 | 0.0000 | **0.0000** |  | 0.0130 | 0.0254 | 0.1151 | 0.0673 | 0.0000 | **0.0000** |
| CD8+ Tem | 0.0530 | 0.0653 | 0.1654 | 0.0406 | 0.0011 | **0.0366** |  | 0.0534 | 0.0630 | 0.4353 | 0.0649 | 0.0000 | **0.0000** |  | 0.0289 | 0.0548 | 0.1654 | 0.0843 | 0.0000 | **0.0000** |
| CD8+ naive T-cells | 0.0338 | 0.0533 | 0.0390 | 0.0401 | 0.7034 | 1.0000 |  | 0.0404 | 0.0541 | 0.0416 | 0.0395 | 0.4396 | 1.0000 |  | 0.0402 | 0.0402 | 0.0250 | 0.0323 | 0.1584 | 1.0000 |
| Class-switched memory B-cells | 0.0475 | 0.0301 | 0.0646 | 0.0305 | 0.3246 | 1.0000 |  | 0.0501 | 0.0320 | 0.2074 | 0.0834 | 0.0000 | **0.0000** |  | 0.0478 | 0.0280 | 0.0834 | 0.0501 | 0.0179 | 0.6101 |
| DC | 0.0642 | 0.0313 | 0.1361 | 0.0453 | 0.0004 | **0.0122** |  | 0.0429 | 0.0470 | 0.0000 | 0.0000 | 0.0000 | **0.0000** |  | 0.0631 | 0.0432 | 0.1337 | 0.0460 | 0.0000 | **0.0007** |
| Eosinophils | 0.0326 | 0.0336 | 0.0344 | 0.0785 | 0.4340 | 1.0000 |  | 0.0297 | 0.0419 | 0.9768 | 0.1262 | 0.0000 | **0.0000** |  | 0.0674 | 0.0642 | 0.0827 | 0.1544 | 0.3795 | 1.0000 |
| Macrophages | 0.0164 | 0.0234 | 0.0511 | 0.0256 | 0.0100 | 0.3393 |  | 0.0225 | 0.0291 | 0.0000 | 0.0000 | 0.0000 | **0.0000** |  | 0.0266 | 0.0469 | 0.0472 | 0.0432 | 0.0097 | 0.3282 |
| Macrophages M1 | 0.0012 | 0.0023 | 0.0170 | 0.0165 | 0.0003 | **0.0101** |  | 0.0050 | 0.0117 | 0.0000 | 0.0000 | 0.0008 | **0.0287** |  | 0.0012 | 0.0047 | 0.0078 | 0.0196 | 0.2758 | 1.0000 |
| Macrophages M2 | 0.0161 | 0.0179 | 0.0355 | 0.0246 | 0.0394 | 1.0000 |  | 0.0385 | 0.0261 | 0.0000 | 0.0000 | 0.0000 | **0.0000** |  | 0.1387 | 0.0629 | 0.1322 | 0.0590 | 0.3762 | 1.0000 |
| Mast cells | 0.0019 | 0.0032 | 0.0087 | 0.0099 | 0.0431 | 1.0000 |  | 0.0049 | 0.0111 | 0.0661 | 0.0091 | 0.0000 | **0.0000** |  | 0.0047 | 0.0047 | 0.0121 | 0.0105 | 0.0159 | 0.5390 |
| Memory B-cells | 0.0060 | 0.0120 | 0.0429 | 0.0439 | 0.0047 | 0.1604 |  | 0.0132 | 0.0192 | 0.0000 | 0.0000 | 0.0000 | **0.0013** |  | 0.0040 | 0.0080 | 0.0397 | 0.0423 | 0.0004 | **0.0127** |
| Monocytes | 0.0348 | 0.0337 | 0.1717 | 0.0499 | 0.0000 | **0.0017** |  | 0.0109 | 0.0224 | 0.2485 | 0.0412 | 0.0000 | **0.0000** |  | 0.0440 | 0.0439 | 0.1476 | 0.0574 | 0.0000 | **0.0002** |
| NK cells | 0.0045 | 0.0155 | 0.0645 | 0.0410 | 0.0001 | **0.0031** |  | 0.0074 | 0.0153 | 0.2163 | 0.0732 | 0.0000 | **0.0000** |  | 0.0053 | 0.0146 | 0.0423 | 0.0431 | 0.0001 | **0.0035** |
| NKT | 0.1263 | 0.0667 | 0.1380 | 0.0540 | 0.7119 | 1.0000 |  | 0.0411 | 0.0415 | 0.9822 | 0.1027 | 0.0000 | **0.0000** |  | 0.1796 | 0.0921 | 0.1662 | 0.1345 | 0.3762 | 1.0000 |
| Neutrophils | 0.0268 | 0.0270 | 0.0905 | 0.0129 | 0.0001 | **0.0027** |  | 0.0447 | 0.0361 | 0.4962 | 0.0225 | 0.0000 | **0.0000** |  | 0.0254 | 0.0294 | 0.0835 | 0.0202 | 0.0000 | **0.0000** |
| Plasma cells | 0.0386 | 0.0235 | 0.0478 | 0.0449 | 0.8535 | 1.0000 |  | 0.0301 | 0.0297 | 0.0000 | 0.0000 | 0.0000 | **0.0000** |  | 0.0294 | 0.0237 | 0.0394 | 0.0422 | 0.8135 | 1.0000 |
| Tgd cells | 0.0111 | 0.0213 | 0.0392 | 0.0327 | 0.0219 | 0.7460 |  | 0.0256 | 0.0301 | 0.2101 | 0.0372 | 0.0000 | **0.0000** |  | 0.0101 | 0.0214 | 0.0555 | 0.0470 | 0.0002 | **0.0079** |
| Th1 cells | 0.0473 | 0.0404 | 0.0320 | 0.0570 | 0.2044 | 1.0000 |  | 0.0495 | 0.0472 | 0.0132 | 0.0178 | 0.0155 | 0.5283 |  | 0.1218 | 0.0513 | 0.0731 | 0.0688 | 0.0116 | 0.3951 |
| Th2 cells | 0.0217 | 0.0249 | 0.0126 | 0.0154 | 0.4221 | 1.0000 |  | 0.0087 | 0.0210 | 0.0003 | 0.0011 | 0.1427 | 1.0000 |  | 0.0107 | 0.0244 | 0.0218 | 0.0234 | 0.0793 | 1.0000 |
| Tregs | 0.0216 | 0.0278 | 0.0025 | 0.0083 | 0.0345 | 1.0000 |  | 0.0426 | 0.0385 | 0.0377 | 0.0245 | 0.8506 | 1.0000 |  | 0.0310 | 0.0272 | 0.0099 | 0.0321 | 0.0014 | **0.0478** |
| aDC | 0.0983 | 0.0539 | 0.1718 | 0.0328 | 0.0017 | 0.0577 |  | 0.0801 | 0.0557 | 0.0000 | 0.0000 | 0.0000 | **0.0000** |  | 0.0823 | 0.0574 | 0.1647 | 0.0394 | 0.0000 | **0.0010** |
| cDC | 0.0089 | 0.0145 | 0.1033 | 0.0636 | 0.0001 | **0.0024** |  | 0.0318 | 0.0490 | 0.0000 | 0.0000 | 0.0000 | **0.0004** |  | 0.0170 | 0.0266 | 0.0922 | 0.0621 | 0.0000 | **0.0005** |
| iDC | 0.0268 | 0.0200 | 0.0300 | 0.0346 | 0.7818 | 1.0000 |  | 0.0169 | 0.0203 | 0.0000 | 0.0000 | 0.0000 | **0.0001** |  | 0.0598 | 0.0276 | 0.0612 | 0.0379 | 0.7170 | 1.0000 |
| naive B-cells | 0.0031 | 0.0068 | 0.0314 | 0.0385 | 0.0249 | 0.8472 |  | 0.0057 | 0.0121 | 0.0076 | 0.0174 | 0.8798 | 1.0000 |  | 0.0003 | 0.0011 | 0.0296 | 0.0408 | 0.0005 | **0.0176** |
| pDC | 0.0057 | 0.0117 | 0.0464 | 0.0199 | 0.0001 | **0.0048** |  | 0.0130 | 0.0122 | 0.0000 | 0.0000 | 0.0000 | **0.0000** |  | 0.0270 | 0.0265 | 0.0773 | 0.0355 | 0.0000 | **0.0005** |
| pro B-cells | 0.0066 | 0.0090 | 0.0122 | 0.0178 | 0.4340 | 1.0000 |  | 0.0096 | 0.0115 | 0.0000 | 0.0000 | 0.0000 | **0.0001** |  | 0.0144 | 0.0141 | 0.0299 | 0.0397 | 0.3066 | 1.0000 |
| NR; no rejection; AMR. Antibody-mediated rejection; aP values obtained by the Mann-Whitney U test. In parentheses is the corrected p-value (pc) using the Bonferroni method for multiple comparisons. Significant results are marked in bold. Values of pc<0.05 were considered significant. | | | | | | | | | | | | | | | | | | | | |
