# Supplementary material for: Computational Prediction of Biomarkers, Pathways, and New Target Drugs in the Pathogenesis of Immune-Based Diseases Regarding Kidney Transplantation Rejection
Source: Front Immunol. 2021 Dec 15;12:800968. doi: 10.3389/fimmu.2021.800968 (PMC8714745; doi:10.3389/fimmu.2021.800968)
Supplement: Supplementary file 3 [file Table_3.docx]

| **Table S3.** The principal KEGG metabolic pathway analysis in DEGs. | | | |
| --- | --- | --- | --- |
| **A. Over-expressed DEGs in the AMR group** | | | |
| **Route ID** | **Term** | **ER** | **FDR^a^** |
| **hsa05150** | *Staphylococcus aureus* infection | 11.59 | <0.0001 |
| **hsa05332** | Graft versus host disease (GVHD) | 10.89 | <0.0001 |
| **hsa05330** | Allograft rejection | 9.61 | <0.0001 |
| **hsa04940** | Type I diabetes mellitus | 9.44 | <0.0001 |
| **hsa05140** | Leishmaniasis | 9.32 | <0.0001 |
| **B. Under-expressed DEGs in the AMR group** | | | |
| **Route ID** | **Término** | **ER** | **FDR^a^** |
| **hsa00071** | Fatty acid degradation | 63.65 | 0.0022 |
| **hsa00280** | Isoleucine, Leucine, and Valine Degradation | 58,35 | 0.0022 |
| AMR, Antibody-mediated rejection; DEGs, Differentially expressed genes; FDR, False Discovery Rate; ER, Enrichment ratio.  ^a^FDR values <0.05 were considered significant. | | | |
