# Supplementary material for: Computational Prediction of Biomarkers, Pathways, and New Target Drugs in the Pathogenesis of Immune-Based Diseases Regarding Kidney Transplantation Rejection
Source: Front Immunol. 2021 Dec 15;12:800968. doi: 10.3389/fimmu.2021.800968 (PMC8714745; doi:10.3389/fimmu.2021.800968)
Supplement: Supplementary file 4 [file Table_4.docx]

| **Table S4.** Protein kinases associated with the different substrates encoded by differentially expressed genes | | | |
| --- | --- | --- | --- |
| **A. Kinases associated with substrates encoded by genes over-expressed in the AMR group** | | | |
| **Kinases** | **Substrates** | **p^a^** |  |
| **MAPK3** | SPIB, MYC, AKT1, MED1, RUNX1, GATA1, EGR1, JUN, CDKN1A, MED14, NCOA1, FOS, TAL1, PPARG, RXRA, STAT1, STAT3, NR3C1, PGR, SMAD3, SP1, CDK1, GSK3B, CEBPA, CEBPB, MAPK8, POLR2A, KLF4, PML, STUB1 | <0.0001 |  |
| **CSNK2A1** | SPI1, AKT1, DAXX, CSNK2A1, JUN, EZH2, FOS, SIRT1, PPARG, STAT1, STAT3, SQSTM1, FUS, HSP90AA1, GADD45GIP1, STUB1, TRIM28, EGR1, SMARCA4, NCOA3, RELA, NPM1, VDR, TCF3, GSK3B, IRF1, RB1, MYC, MED1, HIF1A, CDKN1A, NCOR2, NCOR1, TLE1, PGR, DNMT1, CDK1, SIN3A, CEBPB, HDAC2, HDAC3, KLF4, PML, RUNX1, SUZ12, SMAD3, HSPA4, MAPK9 | <0.0001 |  |
| **MAPK14** | AKT1, JUN, EZH2, FOS, STAT1, STAT3, SQSTM1, FUS, SP1, NFE2L2, HSP90AA1, EGR1, SMARCA4, NCOA3, RELA, NPM1, NR3C1, TCF3, GSK3B, RB1, MYC, HIF1A, CDKN1A, NCOR2, NCOR1, TLE1, PGR, DNMT1, CDK1, SIN3A, CEBPA, CEBPB, POLR2A, KLF4, KAT5, RUNX1, SUZ12, SMAD3, MAPK8 | <0.0001 |  |
| **MAPK1** | RB1, MYC, AKT1, MED1, CSNK2A1, GATA1, HIF1A, JUN, EZH2, CDKN1A, FOS, NCOR2, PPARG, STAT1, STAT3, TLE1, PGR, SP1, CDK1, CEBPA, CEBPB, POLR2A, KLF4, PML, RUNX1, EGR1, NCOA1, RELA, RXRA, NR3C1, SMAD3, TCF3, GSK3B, MAPK8 | <0.0001 |  |
| **ERK1** | SPIB, KAT5, MYC, PRKCD, SQSTM1, GATA1, HIF1A, JUN, SMAD3, NFKB1, SP1, TCF3, FOS, NCOR2, TAL1, CEBPB, RELA, MAPK8, STAT1, STAT3 | <0.0001 |  |
| **B. Kinases associated with substrates encoded by under-expressed genes in the AMR group** | | | |
| **DNAPK** | SOX2, KAT5, PRKCD, PRKDC, SMAD2, SMAD4, SP1, TCF3, HSP90AA1, PARP1, RELA, MAPK8, USF1 | <0.0001 |  |
| **MAPK14** | PPARGC1A, SP1, HSP90AA1, RELA, TCF3, VHL, USF1, RB1, MYC, PRKDC, NCOR1, GTF2I, TLE1, KLF4, KAT5, SKP2, PARP1, SMAD4, SMAD3, MAPK8 | <0.0001 |  |
| **ERK1** | KAT5, MYC, GTF2I, PRKCD, SMAD2, SMAD4, SMAD3, SP1, TCF3, RELA, MAPK8 | <0.0001 |  |
| **MAPK1** | RB1, MYC, GTF2I, TLE1, SP1, KLF4, SKP2, NCOA1, PARP1, RELA, SMAD2, SMAD4, SMAD3, TCF3, MAPK8 | <0.0001 |  |
| **CDK1** | PPARGC1A, SP1, HSP90AA1, RELA, TCF3, IRF3, VHL, USF1, RB1, MYC, PRKDC, NCOR1, GTF2I, HDAC2, KLF4, SOX2, KAT5, SKP2, SMAD4, SMAD3, MAPK8 | <0.0001 |  |
| ^a^P values<0.05 were considered significant. | | | |
