# Supplementary material for: Computational Prediction of Biomarkers, Pathways, and New Target Drugs in the Pathogenesis of Immune-Based Diseases Regarding Kidney Transplantation Rejection
Source: Front Immunol. 2021 Dec 15;12:800968. doi: 10.3389/fimmu.2021.800968 (PMC8714745; doi:10.3389/fimmu.2021.800968)
Supplement: Supplementary file 5 [file Table_5.docx]

| **Table S5.** Transcription factors associated with differentially expressed genes in the AMR group. | | |
| --- | --- | --- |
| **A. Top-5 factors associated with over-expressed genes in the AMR group** | | |
| **TF** | **Target genes** | ***p*^a^** |
| **SPI1** | *GMFG, RNASE6, LYN, FLI1, ARHGAP9, DUSP5, EMP3, PELI1, HCST, PTAFR, PSMB8, CRLF3, NCF2, MAP3K8, S100A4, LY96, CELF2, ITGB2, ITGAL, HHEX, TAGAP, ACTR2, LCP2, LCP1, BIRC3, IKZF1, ICAM1, PIK3R5, CTSS, CD53, GIMAP6, TYROBP, DAPP1, ISG20, PLEK, TNFAIP3, BCL2A1, CYTH4, GNG2, CFP, NCKAP1L, ARPC1B, HCLS1, FGD2, ANKRD44, CSF1R, SLC15A3, CD300LF, EFHD2, LST1, B2M, SP110, INPP5D, AIF1, CD300A, ELMO1, CSF2RB, STK4, ARHGAP25, ARHGAP30, RGS10* | <0.0001 |
| **IRF8** | *EPSTI1, TAP2, TAP1, CD274, DAPP1, SLC15A3, MLKL, LY86, PARP14, ISG20, RTP4, CIITA, SECTM1, CTSS, TNFSF13B, CASP1, CD53, TLR4, AIF1, PSMB10, HLA-DMB, GBP2, PSMB8, PSMB9* | <0.0001 |
| **RUNX1** | *GMFG, STARD4, RNASE6, ARHGAP9, TMC8, EMP3, HCST, BID, IL12RB1, SLA, STK10, MAP3K8, MFNG, CELF2, ITGB2, ITGAL, ITGA4, LCP2, LCP1, FYB, IFI16, SH2B3, MYO1F, PRF1, IKZF1, LDLRAD4, RASSF5, LOC153684, CCR2, FCER1G, CD48, SELPLG, VEGFC, CD69, GRK5, TNFAIP3, SAMHD1, CYTH4, LYST, PLEKHO2, ADCY7, APOL6, IL10RA, SLC15A3, PECAM1, P2RX7, EVI2A, EVI2B, SH2D1B, GLIPR1, TNFAIP8L2, IL18RAP, CD300A, ELMO1, CSF2RB, STK4, ARHGAP30, FGR, RGS10* | <0.0001 |
| **RELA** | *RCSD1, STAT1, PSMB8, NCF2, MAP3K8, CIITA, ITGB2, LCP1, BIRC3, IL2RG, SH2B3, MARCKS, MYO1G, ICAM1, B2M, PLA1A, EBI3, BST2, CD48, STX11, CD69, PMAIP1, TNFAIP3, NFKBIZ, SRGN, FCHSD2, AIM2, IRF1* | <0.0001 |
| **IRF1** | *UBE2L6, B2M, PRR11, APOBEC3C, PARP14, C5ORF56, WARS, VAMP5, APOL6, STAT1, PSMB8, SRGN, TNFRSF1B, PSMB10* | <0.0001 |
| **B. Top-5 of transcription factors associated with under-expressed genes in the AMR group** | | |
| **MYC** | *TNPO2, SLC25A39, ATP5B, PTDSS2* | 0.014 |
| **KLF4** | *EPB41L1, SLC25A23, SLC25A39, ACADS, SH3D21* | 0.022 |
| **USF1** | *C12ORF49, TEF, TIGD5, TNPO2, ALDH3A2, POLDIP2* | 0.027 |
| **SOX2** | *EPB41L1, DNAJC6, ZCCHC14, TMED4* | 0.038 |
| **TCF3** | *SLC25A39, ATP5B, TIGD5, POLDIP2* | 0.049 |
| ^a^P values<0.05 were considered significant. TF, transcription factors. AMR, Antibody-mediated rejection. | | |
